# Comparative genomic insights into habitat adaptation of coral-associated *Prosthecochloris*

**DOI:** 10.3389/fmicb.2023.1138751

**Published:** 2023-04-20

**Authors:** Zhaolong Nie, Kaihao Tang, Weiquan Wang, Pengxia Wang, Yunxue Guo, Yan Wang, Shuh-Ji Kao, Jianping Yin, Xiaoxue Wang

**Affiliations:** ^1^State Key Laboratory of Marine Resource Utilization in South China Sea, Hainan University, Haikou, China; ^2^Key Laboratory of Tropical Marine Bio-resources and Ecology, Guangdong Key Laboratory of Marine Materia Medica, Innovation Academy of South China Sea Ecology and Environmental Engineering, South China Sea Institute of Oceanology, Chinese Academy of Sciences, Guangzhou, China; ^3^Southern Marine Science and Engineering Guangdong Laboratory (Guangzhou), Guangzhou, China; ^4^University of Chinese Academy of Sciences, Beijing, China

**Keywords:** *Prosthecochloris*, coral skeleton, endolithic bacteria, adaptive strategy, mobile genetic elements

## Abstract

Green sulfur bacteria (GSB) are a distinct group of anoxygenic phototrophic bacteria that are found in many ecological niches. *Prosthecochloris*, a marine representative genus of GSB, was found to be dominant in some coral skeletons. However, how coral-associated *Prosthecochloris* (CAP) adapts to diurnal changing microenvironments in coral skeletons is still poorly understood. In this study, three *Prosthecochloris* genomes were obtained through enrichment culture from the skeleton of the stony coral *Galaxea fascicularis*. These divergent three genomes belonged to *Prosthecochloris marina* and two genomes were circular. Comparative genomic analysis showed that between the CAP and non-CAP clades, CAP genomes possess specialized metabolic capacities (CO oxidation, CO_2_ hydration and sulfur oxidation), gas vesicles (vertical migration in coral skeletons), and *cbb*_3_-type cytochrome *c* oxidases (oxygen tolerance and gene regulation) to adapt to the microenvironments of coral skeletons. Within the CAP clade, variable polysaccharide synthesis gene clusters and phage defense systems may endow bacteria with differential cell surface structures and phage susceptibility, driving strain-level evolution. Furthermore, mobile genetic elements (MGEs) or evidence of horizontal gene transfer (HGT) were found in most of the genomic loci containing the above genes, suggesting that MGEs play an important role in the evolutionary diversification between CAP and non-CAP strains and within CAP clade strains. Our results provide insight into the adaptive strategy and population evolution of endolithic *Prosthecochloris* strains in coral skeletons.

## 1. Introduction

The coral animal and its associated zooxanthella, bacteria, archaea, fungi, and viruses, collectively constitute the coral holobiont (Rosenberg et al., 2007; Bourne et al., 2016). Microorganisms inhabiting coral surface mucus, tissue and gastric cavity play different roles in coral health, including nutrient cycling, and antimicrobial and antioxidant activities (Morris et al., 2019; Peixoto et al., 2021; Cardenas et al., 2022). Similarly, a variety of algae, fungi, bacteria and microeukaryotes inhabit coral skeletons (Li et al., 2014; Yang et al., 2016; Marcelino et al., 2018; Cardenas et al., 2022), and some groups of endolithic microorganisms are also involved in the nutrient cycling and metabolite transfer of coral holobionts (Pernice et al., 2020). *Prosthecochloris,* green sulfur bacteria (GSB) capable of anoxygenic photosynthesis and nitrogen fixation, was dominant in some layers of coral skeletons or whole coral samples in different corals (Yang et al., 2016, 2019; Cai et al., 2017; Yu et al., 2021; Chen et al., 2021b).

*Prosthecochloris* are regarded as typical marine representatives of GSB, which represent one phylum Chlorobiota, with one family Chlorobiaceae, including the genera *Chlorobium*, *Chlorobaculum*, *Prosthecochloris* and *Chloroherpeton* (Imhoff, 2014; Oren and Garrity, 2021). GSB are a distinct group of strictly anaerobic and anoxygenic phototrophic bacteria with specialized photosynthetic reactions and metabolic properties of carbon fixation and nitrogen fixation (Imhoff, 2014). GSB possess light-harvesting complexes called chlorosomes which contain a large number of special bacteriochlorophylls. Chlorosomes are highly efficient and can capture minute amounts of light, which enables bacteria to adapt to low-light conditions (Imhoff, 2014). GSB have been in many marine ecological niches with variable light availability (Imhoff, 2014) including special photosynthetic habitats such as coral skeletons (Li et al., 2014; Yang et al., 2016) and deep-sea hydrothermal vents (Beatty et al., 2005). GSB are obligately phototrophic and strictly dependent on photosynthesis under anoxic conditions, using inorganic electron donors and fixing carbon dioxide through reductive citrate cycle. Sulfide is an important photosynthetic electron donor in GSB and the oxidation of sulfide usually deposits elemental sulfur globules outside the cells. This process enables syntrophic associations between GSB and sulfur- and sulfate-reducing bacteria (Imhoff, 2014). Species of *Prosthecochloris* possess all the above features of GSB and are nonmotile (Gorlenko, 2015). *Prosthecochloris* spp. differ in cell shapes, spherical or ovoid, and have different requirements for vitamin B_12_ as a growth factor (Anil Kumar et al., 2009; Gorlenko, 2015; Bryantseva et al., 2019)_._ *Prosthecochloris* spp. were found in diverse environments including hydrogen sulfide-rich mud, hot spring sediment and coral skeletons (Gorlenko, 1970; Imhoff, 2003; Beatty et al., 2005; Nabhan et al., 2016; Cai et al., 2017; Thiel et al., 2017; Grouzdev et al., 2018; Bryantseva et al., 2019; Yang et al., 2019; Kyndt et al., 2020; Athen et al., 2021; Chen et al., 2021a,b). The major difference between coral-associated and other non-coral-associated *Prosthecochloris* remains poorly understood, although preliminary analysis of coral-associated *Prosthecochloris* was performed previously (Chen et al., 2021b).

The microniches within the skeleton can be shaped by physico-chemical gradients and diurnal rhythms (Shashar and Stambler, 1992; Ricci et al., 2019). In the daytime, sunlight is mostly depleted by coral, and only small amounts of light reach the skeleton (Ricci et al., 2019). Meanwhile, the O_2_ concentration is gradually decreased from the vicinity of tissue to the inside of skeleton but with a high O_2_ concentration in the endolithic algal zone due to photosynthesis (Ricci et al., 2019). At night, the O_2_ concentration in the skeleton is greatly decreased because of the dominance of heterotrophic metabolism during the night (Ricci et al., 2019). Accordingly, the pH is high up to 8.5 in the daytime and low at night (Shashar and Stambler, 1992). How endolithic *Prosthecochloris* adapts to these changing microenvironments in coral skeletons is not well understood.

*Galaxea fascicularis* is a massive stony coral belonging to the family Oculinidae, having large connected coenosteum with skeletal vesicles varying from 0.2 to 0.5 mm (Wewengkang et al., 2007; Hou et al., 2018). In this study, *Prosthecochloris* was found in the skeleton of *G. fascicularis*, and three *Prosthecochloris* genomes were obtained through enrichment culture. Two *Prosthecochloris* genomes were circular. Comprehensive comparative genomic analysis was preformed and the genomic features of CAP genomes were revealed. Between CAP and non-CAP genomes, CAP genomes possess specialized metabolic capacities and genes coding gas vesicle proteins and *cbb*_3_-type cytochrome *c* oxidases. Within CAP clade genomes, polysaccharide synthesis gene clusters and phage defense systems may endow bacteria with differential cell surface structures and phage susceptibility, driving strain-level evolution. Our results provide insight into the adaptive strategy of *Prosthecochloris* strains to thrive in ecological niches such as coral skeletons.

## 2. Materials and methods

### 2.1. Sample collection and enrichment culture

Skeleton samples were obtained from *G. fascicularis* fragments used in our previous study (Wang et al., 2022). Medium for enrichment culture was prepared according to previous studies (Zyakun et al., 2009; Yang et al., 2019), with additional glucose (0.05%) and resazurin (1 μg L^−1^) as a redox indicator. Skeleton samples were rapidly added to filter sterilized medium in Hungate anaerobic tubes and cultured at room temperature (~ 25–28°C) under natural sunlight. After approximately 2 weeks, green colors were visible in the two enrichment cultures (with or without resazurin) and cells were examined by microscopy and collected by centrifugation for DNA extraction.

### 2.2. DNA extraction, genome sequencing, and assembly

Total DNA of collected cells was isolated using the TIANamp Bacteria DNA kit (Tiangen Biotech Co. Ltd., Beijing, China) and sequenced using PacBio and IlIumina platforms. Metagenome assembly and binning were performed using MetaWRAP pipeline v1.3.2 (Uritskiy et al., 2018) with wrapped tools of SPAdes 3.13.0 (Bankevich et al., 2012), MaxBin2 v2.2.7 (Wu et al., 2016), CONCOCT v1.0.0 (Alneberg et al., 2014) and metaBAT2 v2.12.1 (Kang et al., 2019). The qualities of metagenomic bins were accessed by CheckM v1.2.1 (Parks et al., 2015). The percentage of reads mapped to each bin and proportion of a bin relative to all recovered bins were evaluated by CheckM. Classification of metagenomic bins was performed by GTDB-TK v2.1.1 (Chaumeil et al., 2022). In order to obtain complete genomes belonging to *Prosthecochloris*, long reads were used to assemble genomes by HGAP v4 within SMRT Link, and polished by pilon (Walker et al., 2014) and homopolish (Huang et al., 2021). Pairwise ANI between genomes calculations were performed using FastANI (Jain et al., 2018). Phylogenetic analysis of 16S rRNA genes was performed using MEGA (Kumar et al., 2016) and phylogenetic analysis of whole genomes based on core gene was performed using FastTree (Price et al., 2009).

### 2.3. 16S rRNA gene amplicon sequencing

The barcoded primers 515 (Parada) (5’-GTGYCAGCMGCCGCGGTAA-3′)/806 (Apprill) (5’-GGACTACNVGGGTWTCTAAT-3′) were used to amplify the V4 region of microbial 16S rRNA (Apprill et al., 2015; Parada et al., 2016). The purified PCR products were sequenced on the Illumina platform. Quality-filtered reads were submitted to the MicrobiomeAnalyst platform (Chong et al., 2020) using DADA2 pipeline (Callahan et al., 2016) against taxonomy reference database of Silva (version 138.1; Yilmaz et al., 2014). A total of 129,228 sequences from two samples corresponding to 55 unique ASVs were recovered. Taxa abundance profiling was performed on the MicrobiomeAnalyst platform with default parameters (Chong et al., 2020).

### 2.4. Comparative genomic analysis

All available *Prosthecochloris* genomes (Table 1) and two *Chlorobium* genomes were downloaded from NCBI and reannotated by Prokka (Seemann, 2014). The highly divergent rates among different protein families may affect the accuracies of protein homolog detection methods, therefore, it is more reliable to combine different methods to identify protein homologs for comparative genomic analysis. Briefly, KEGG orthologs in genomes were identified by BlastKOALA (Kanehisa et al., 2016) and KofamKOALA (Aramaki et al., 2020) online.^1^ Pathway comparisons were performed by KEMET with default parameters (Palu et al., 2022). Pfam domains (Mistry et al., 2021) of proteins were further analyzed by Interproscan (v5.54–87.0) (Jones et al., 2014) with in a local searching mode. Pangenome analysis was performed using Roary (Page et al., 2015) with the following parameter: -I 70. Core gene alignment generated by Roary was used for phylogenetic analysis by FastTree (Price et al., 2009). Furthermore, Scoary (Brynildsrud et al., 2016) was used to explore potential specific genes that were missed in the KEGG pathway comparison. The protein homologs identified by all the above methods were used as a cross validation to obtain a reliable gene presence and absence analysis. Meanwhile, visualization of multiple genome alignments was performed by Mauve (Darling et al., 2004, 2010) and Proksee with its built-in tools (Vernikos and Parkhill, 2006; Couvin et al., 2018; Guo et al., 2021; Brown et al., 2022). A Venn diagram was drawn by jvenn (Bardou et al., 2014). GO enrichment of genes was performed by TBtools (Chen et al., 2020).

**Table 1:** Information of *Prosthecochloris* genomes assembled from pure cultures, enrichment cultures or metagenomes.

Strain	Contig	CDS	Size (Mb)	GC%	Source^b^	Isolation	Reference^c^
*Prosthecochloris* sp. Ty-1 (=TY Vent = GSB1)	1	2,270	2.47	56	PC	Deep-sea hydrothermal vent	Beatty et al. (2005)
*Candidatus* Prosthecochloris sp. C10	41	1,953	2.13	49.1	MAG	Seawater lake chemocline	GCA_010912745.1
*Candidatus* Prosthecochloris sp. A305	75	1,999^a^	2.09	47.8	MAG	Skeleton of coral *Isopora palifera*	Yang et al. (2019)
*Candidatus* Prosthecochloris sp. N2	46	2,534^a^	2.65	47.4	EC	Skeleton of coral *Isopora palifera*	Chen et al. (2021b)
*Candidatus* Prosthecochloris korallensis	68	2,485^a^	2.58	48.3	MAG	Skeleton of coral	Cai et al. (2017)
*Candidatus* Prosthecochloris sp. SCSIO W1101	1	2,918^a^	3.02	47.2	EC	Skeleton of coral *Galaxea fascicularis*	This study
*Candidatus* Prosthecochloris sp. N1	24	2,630^a^	2.79	47	EC	Skeleton of coral *Isopora palifera*	Chen et al. (2021b)
*Prosthecochloris marina* V1	19	2,474	2.72	47	PC	South China Sea coastal zone	Bryantseva et al. (2019)
*Candidatus* Prosthecochloris sp. SCSIO W1102	1	1,270^a^	1.32	47.1	EC	Skeleton of coral *Galaxea fascicularis*	This study
*Candidatus* Prosthecochloris sp. SCSIO W1103	1	2,627^a^	2.79	47.1	EC	Skeleton of coral *Galaxea fascicularis*	This study
*Candidatus* Prosthecochloris sp. B10	32	2,007	2.26	50.1	MAG	Seawater lake chemocline	GCA_010912735.1
*Prosthecochloris* sp. ZM	40	2,416	2.66	50	PC	Meromictic lakes Green cape	Grouzdev et al. (2018)
*Prosthecochloris aestuarii* DSM 271	2	2,308	2.58	50.1	PC	Hydrogen sulfide-rich mud	Gorlenko (1970)
*Prosthecochloris vibrioformis* DSM 260	75	2,103	2.31	52.1	PC	Rivermouth	Imhoff (2003)
*Prosthecochloris* sp. CIB 2401	1	2,166	2.40	52.1	PC	Coastal brackish lagoon	Nabhan et al. (2016)
*Candidatus* Prosthecochloris vibrioformis M55B161	8	1,959	2.13	52.4	MAG	Saline lake water	Chen et al. (2021a)
*Candidatus* Prosthecochloris vibrioformis M50B85	18	2,031	2.23	52.2	MAG	Saline lake water	Chen et al. (2021a)
*Prosthecochloris* sp. HL-130-GSB	1	2,152	2.41	52	PC	Microbial mat in Hot Lake	Thiel et al. (2017)
Prosthecochloris sp. SM2_Orange-Green1	112	2,245	2.43	51.8	EC	Inland salt marsh	Athen et al. (2021)
*Candidatus* Prosthecochloris aestuarii SpSt-1,181	228	1,908	2.03	52.2	MAG	Hot spring sediment	GCA_011054385.1
*Prosthecochloris* sp. ZM_2	117	2,203	2.40	55.5	PC	Meromictic lakes Green cape	Grouzdev et al. (2018)
*Prosthecochloris ethylica* DSM 1685	66	2,179	2.44	55.1	PC	Mud sample from estuary	Kyndt et al. (2020)
*Candidatus* Prosthecochloris ethylica N2	60	2,181	2.44	55.1	EC	Lake mud	Kyndt et al. (2020)
*Candidatus* Prosthecochloris ethylica N3	72	2,189	2.45	55.1	EC	Mud sample from estuary	Kyndt et al. (2020)

a Counts of coding sequences (CDS) are from Prokka annotation.

b PC, pure culture; EC, metagenomic assembly of enrichment culture; MAG, metagenome-assembled genomes.

c If no publication was found, genome assembly accessions were provided instead.

## 3. Results and discussion

### 3.1. *Prosthecochloris* cells in enrichment cultures from the skeleton of *Galaxea fascicularis*

**Figure 1:**
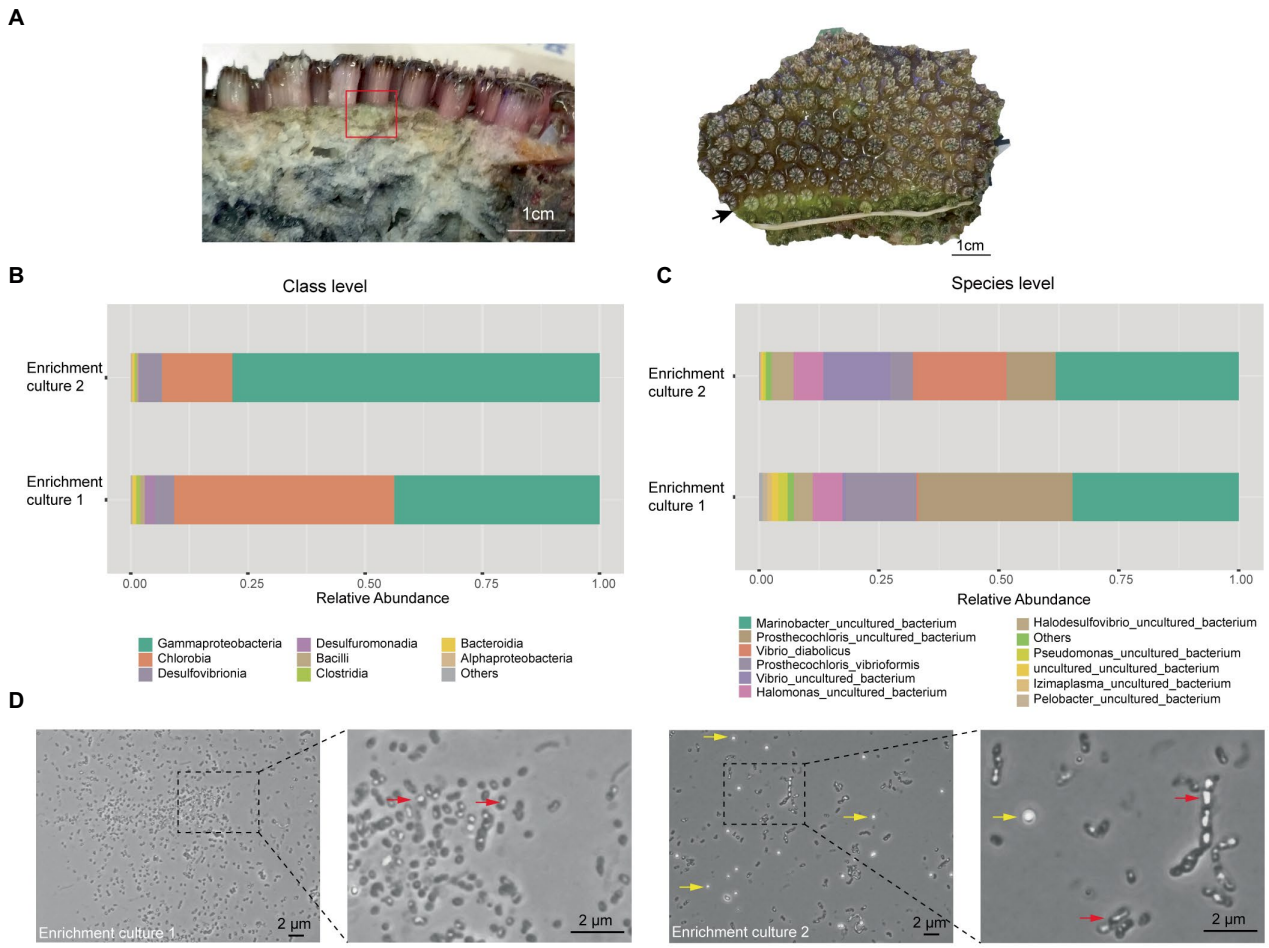
*Prosthecochloris* cells were enriched from the skeleton of *Galaxea fascicularis*. **(A)** Lateral and frontal views of the *Galaxea fascicularis* skeleton. The red rectangle indicates the region of the skeleton sampled for enrichment culture. The black arrow indicates a green layer in the corallites after polyp bleaching. Relative abundance of major microbial taxa in enrichment cultures at class level **(B)** and at species level **(C)**. **(D)** Cell morphology of *Prosthecochloris* cells. Red arrows indicate gas vesicles in cells and yellow arrows indicate sulfur granules outside cells.

During our previous study of the microbiome of the reef-building coral *Galaxea fascicularis* (Wang et al., 2022), a light green layer in the skeleton directly under the coenosarc was observed (Figure 1A left panel), similar to the *Prosthecochloris* formed green layer in the skeleton of *Isopora palifera* (Yang et al., 2019; Chen et al., 2021b). Occasionally, this green layer could cover the corallites after polyp bleaching (Figure 1A right panel). *Prosthecochloris* was found in our previous 16S rRNA gene amplicon sequencing data of *G. fascicularis* polyp samples (Supplementary Figure S1), therefore, we tended to culture *Prosthecochloris* from the skeleton of *G. fascicularis*. We finally obtained two enrichment cultures (with or without the redox indicator resazurin in the medium) from the same skeleton sample (Figure 1A left panel). 16S rRNA gene amplicon sequencing showed that the relative abundance of Chlorobia and Gammaproteobacteria accounted for ~90% of total reads at class level (Figure 1B). Accordingly, two species of *Prosthecochloris* accounted for the majority of Chlorobia, while *Marinobacter*, *Vibrio* and *Halomonas* accounted for the majority of Gammaproteobacteria (Figure 1C). Furthermore, *Halodesulfovibrio* sp., belonging to sulfate-reducing bacteria, was also found in the enrichment cultures (Figure 1C). *Candidatus* Halodesulfovibrio lyudaonia was previously recovered along with *Prosthecochloris* spp. from the same enrichment culture and proposed having a syntrophic relationship with coral-associated *Prosthecochloris* (Chen et al., 2021b). Amplification and sequencing 16S rRNA gene sequences using the 27F/1492R primer pair from total DNA of these two cultures showed both ~99% to *Prosthecochloris marina* V1 (abbreviated as PmV1; Bryantseva et al., 2019). Observation of cells by microscopy revealed many cells with gas vesicles (Figure 1D), a typical feature of *Prosthecochloris* (Bryantseva et al., 2019). Bright granules were observed in enrichment culture 2, highly similar to the extracellular sulfur granules previously observed in *Prosthecochloris indica* (Anil Kumar et al., 2009). Furthermore, some cells were connected in a chain, similar with that cells of *Prosthecochloris aestuarii* were often connected by one or two filaments (Gorlenko, 1970; Gorlenko, 2015). These results indicate that *Prosthecochloris* with other potentially syntrophic bacteria were cultured from the skeleton of *G. fascicularis*.

### 3.2. Complete *Prosthecochloris* genomes recovered from *Galaxea fascicularis* endolithic cultures

**Figure 2:**
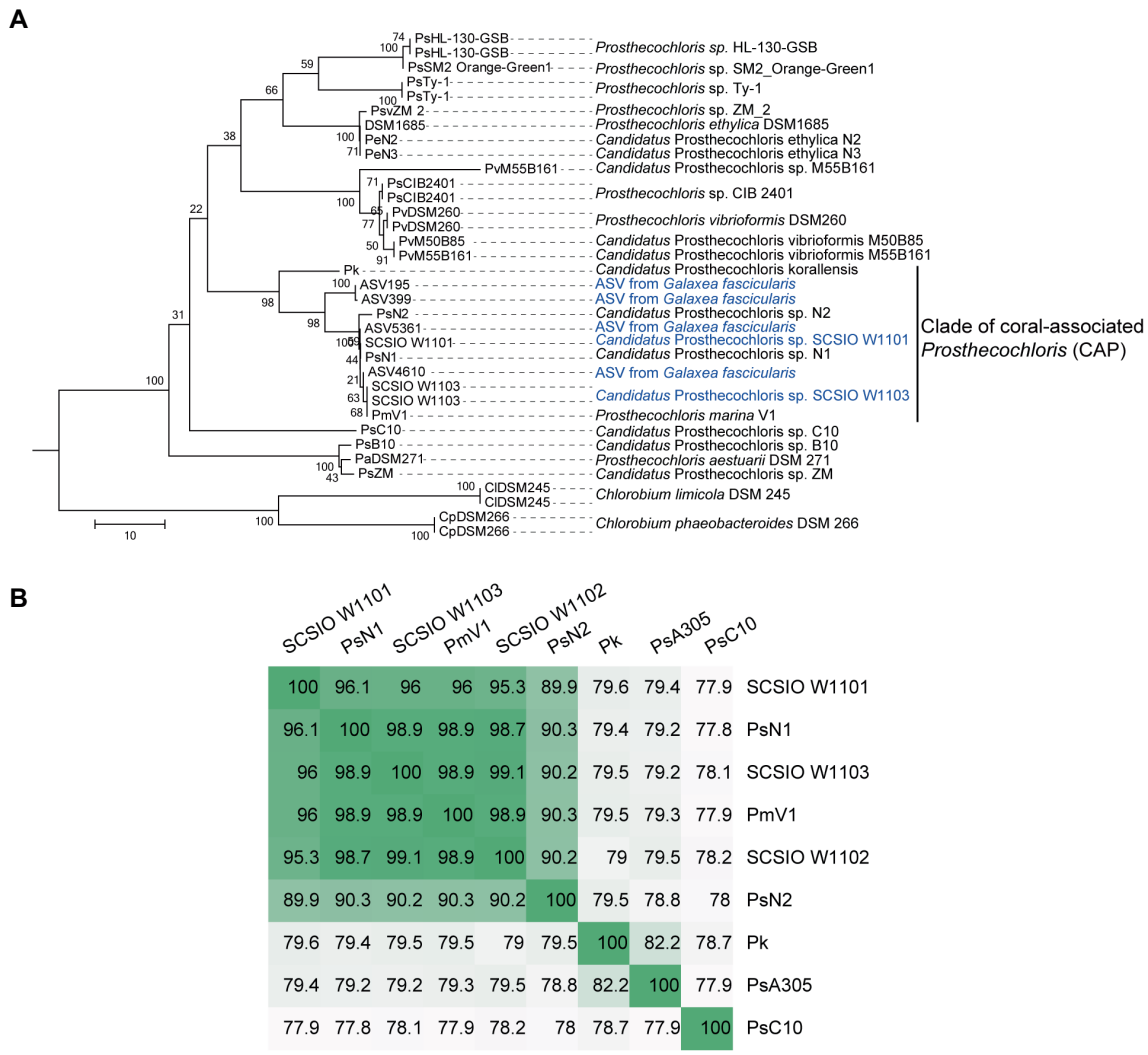
Classification of coral-associated *Prosthecochloris* (CAP) genomes. **(A)** The neighbor-joining tree was constructed using MEGA software with 1,000 bootstraps after aligned by MAFFT. Sequences of 16S rRNA genes of other *Prosthecochloris* genome assemblies listed in Table 1 were extracted. Sequences of amplicon sequence variants (ASVs) were extracted from our previous full-length 16S rRNA gene amplicon sequencing data of *Galaxea fascicularis* samples (Wang et al., 2022). Sequences from two *Chlorobium* strains were used as the outgroup. Branch lengths are proportional to the number of nucleotide substitutions. A short name for each genome assembly is shown at the end of each branch. **(B)** Average nucleotide identity of CAP genomes. *Candidatus Prosthecochloris* sp. C10 (abbreviated as PsC10) is the closest non-CAP clade *Prosthecochloris* genome.

**Figure 3:**
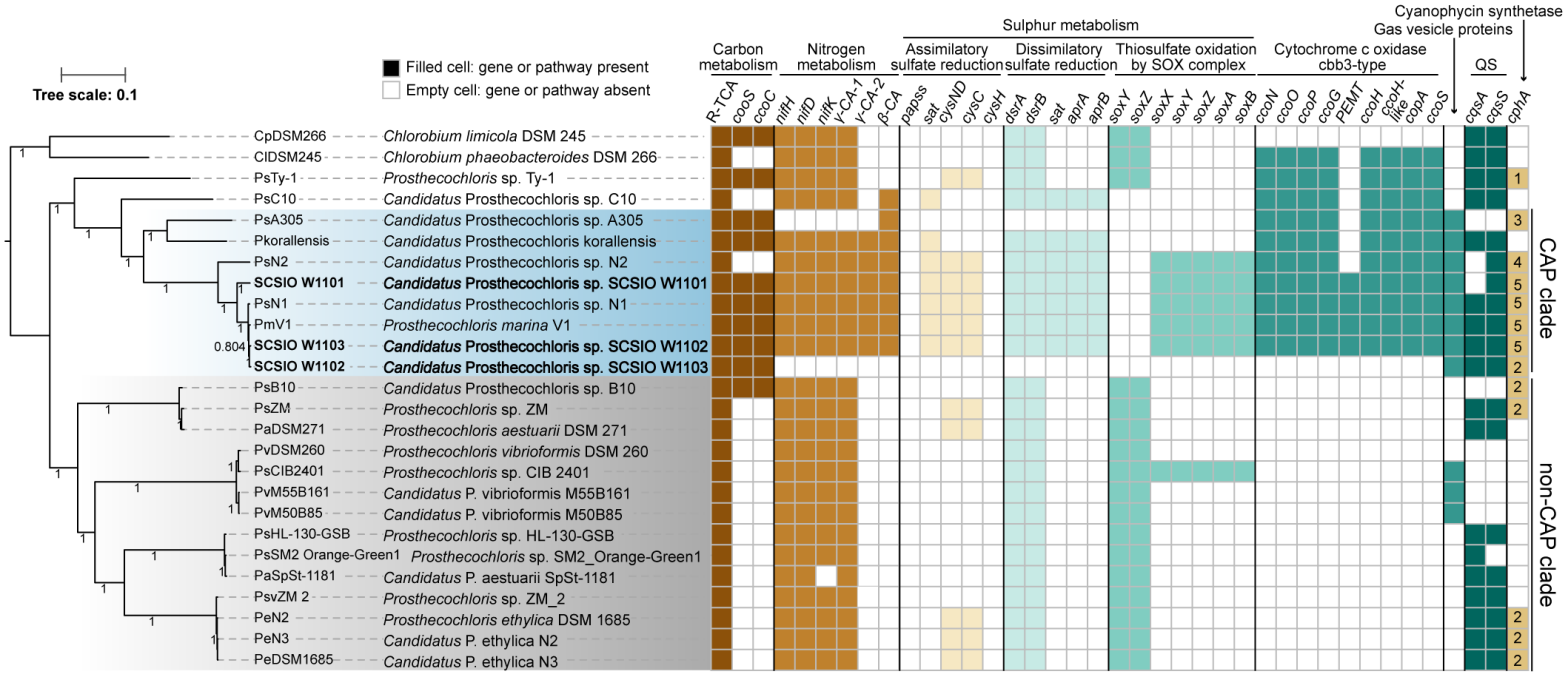
Coral-associated *Prosthecochloris* (CAP) genomes show specialized metabolic capacities. Phylogenetic relationship of *Prosthecochloris* genomes and differences in metabolic capacities. CAP genomes are in blue shading, non-CAP genomes are in gray shading, and the names of new identified *Prosthecochloris* genomes in this study are in bold format. The phylogenetic trees of concatenated genes were constructed by FastTree. Different colors of filled cells indicate different classifications of genes, but not the copy numbers of the genes, except that the numbers in the cells of gene *cphA* indicate the copy numbers. R-TCA, reductive citrate cycle.

In order to obtain the genomes in the enrichment cultures, total DNA of each culture was extracted and sequenced using PacBio and Illumina sequencing platforms. Analysis of metagenome-assembled genomes (MAGs) using short reads revealed that bacterial genomes belonging to *Prosthecochloris*, *Marinobacter*, *Vibrio*, *Halomonas* and *Halodesulfovibrio* were obtained (Supplementary Table S1), consistent with the 16S rRNA gene amplicon sequencing data. *Prosthecochloris*–derived bins accounted for 81 and 58% of total communities of enrichment culture 1 and 2. In order to obtain the complete genomes of *Prosthecochloris*, genomes were reassembled using long and short reads. Finally, one circular genome with 3.02 Mbp (SCSIO W1101) and one circular genome with 2.79 Mbp (SCSIO W1103) were recovered from enrichment cultures 1 and 2, respectively (Table 1). A partial genome with 1.32 Mbp (SCSIO W1102) was also recovered from enrichment culture 1. Analysis using GTDB-Tk (Chaumeil et al., 2022) confirmed that these three genomes belong to the *Prosthecochloris* genus. The 16S rRNA gene sequences in these genomes are nearly identical to the extracted full-length 16S rRNA gene amplicon sequence variants (ASVs) of *G. fascicularis*, suggesting that cultured *Prosthecochloris* cells are indeed from the skeleton of *G. fascicularis* (Supplementary Figure S2). Along with four previous metagenome-assembled *Prosthecochloris* genomes (Table 1), pairwise comparison of 16S rRNA gene sequences showed that the 16S rRNA genes shared high identity (98.5–100%) with each other (Supplementary Figure S2), and they fell into one clade in the phylogenetic tree (Figure 2A), consistent with the previous report (Chen et al., 2021b). Further analysis based on average nucleotide identity (ANI) across whole genome sequences indicated that strains SCSIO W1101, SCSIO W1102, SCSIO W1103, N1 and V1 belong to the same species, namely *Prosthecochloris* marina and SCSIO W1101 is divergent to the other four strains (ANI 95.3–96.1%; Figure 2B). Constantly, this relationship was confirmed by the phylogenetic analysis using concatenated DNA sequence of core genes (Figure 3). Therefore, this clade was designated the coral-associated *Prosthecochloris* (CAP) clade as previously proposed (Chen et al., 2021b). Accordingly, this relationship indicates that individual and chain-like cells in enrichment cultures may be derived from SCSIO W1101 and SCSIO W1103, respectively (Figure 1). Taken together, these results suggest that the *G. fascicularis* endolithic *Prosthecochloris* population is not a monolithic group that may have high genetic diversity, which is consistent with the *Prosthecochloris* population in the skeleton of coral *I. palifera* (Yang et al., 2019; Chen et al., 2021b).

Among these CAP clade genomes, SCSIO W1101 and SCSIO W1103 are the only two complete genome assemblies, and SCSIO W1101 has the largest genome size among all of these *Prosthecochloris* genomes (Table 1; Supplementary Figure S3A). Moreover, these CAP clade genomes have significantly lower G + C content than non-CAP genomes (Supplementary Figure S3B). A trend toward high G + C content in free-living organisms and low G + C content in bacteria living in nutrient-limiting and nutrient-poor environments (e.g., some symbiotic bacteria) was found previously (Mann and Chen, 2010). Therefore, the low G + C content may be an indicator to differentiate CAP and non-CAP genomes.

### 3.3. Coral-associated *Prosthecochloris* genomes show specialized metabolic capacities

**Figure 4:**
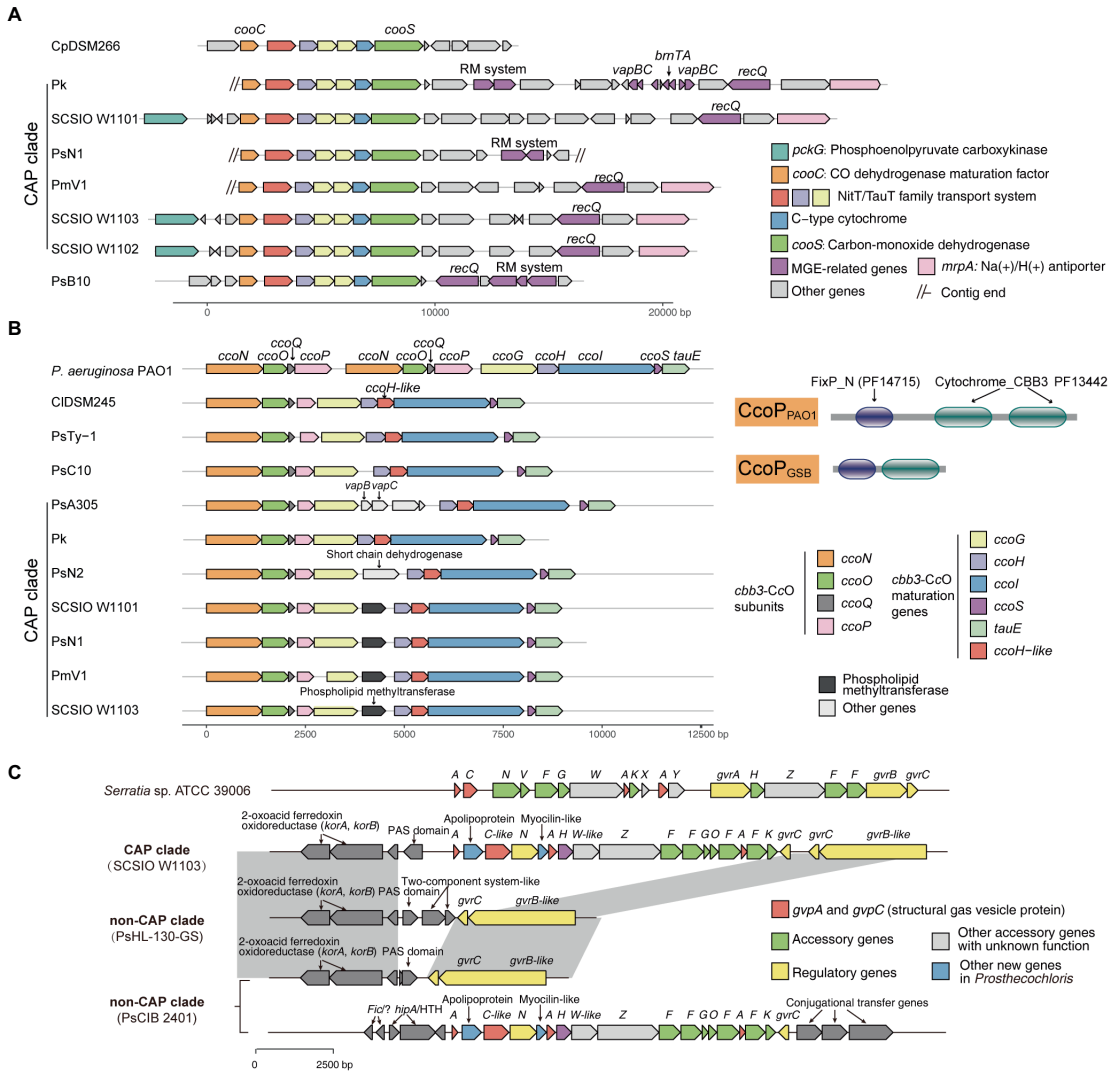
Mobile genetic elements and related genome rearrangements reshape gene clusters involved in the metabolic capacity divergence between coral-associated *Prosthecochloris* (CAP) and non-CAP genomes. **(A)** gene clusters of carbon monoxide dehydrogenase. **(B)** Gene clusters of *cbb*_3_-type cytochrome *c* oxidases (*cbb*_3_-C*c*Os). The domain architectures of different CcoP proteins are shown. PF14715: FixP_N (N-terminal domain of cytochrome oxidase-*cbb*_3_ FixP). PF13442: Cytochrome_CBB3 (Cytochrome C oxidase, *cbb*_3_-type, subunit III). **(C)** Gene clusters of gas vesicle proteins (*gvp*). Gray shading indicates conserved genomic regions.

In order to determine metabolic capacity differences between CAP genomes and non-CAP genomes, comprehensive comparative genomic analysis combining KEGG pathway reconstitution and genome-wide analysis of protein homolog presence or absence was used (see Methods). Phylogenetic analysis using concatenated core genes showed that CAP genomes and non-CAP genomes were separated into different clades (Figure 3). The CAP clade contains 8 genomes belonging to at least 4 species. The non-CAP clade contains 14 genomes belonging to 4 species, i.e., *P. aestuarii*, *P. vibrioformis*, *Prosthecochloris* sp. HL-130-GSB and *P. ethylica*. Generally, the pattern of the presence or absence of several pathways is mostly conserved within the CAP or non-CAP clade, and is different between the CAP and non-CAP clades, although the absence of genes in the CAP clade may be caused by the incompleteness of some MAGs (Figures 3, 4). Moreover, *Prosthecochloris* sp. Ty-1 (abbreviated as PsTy-1) and *Candidatus Prosthecochloris* sp. C10 (abbreviated as PsC10) are more closely related to the CAP clade, and PsC10 shares the last common ancestor with the CAP clade. The metabolic capacity of PsTy-1 or PsC10 is in the intermediate state between the CAP and non-CAP clades.

#### 3.3.1. Production of cobalamin and bacteriochlorophyll in *Prosthecochloris*

Cobalamin (vitamin B_12_) was proposed to be important for coral holobionts. It was found that the concentration of vitamin B_12_ in the coral gastric cavity is high, and bacteria living in the gastric cavity can produce vitamin B_12_ (Agostini et al., 2012). All analyzed GSB contained genes involved in cobalamin biosynthesis pathways (Supplementary Figure S4). In bacteria, anaerobic and aerobic cobalamin biosynthesis pathways share several genes and possess pathway-specific genes. Genes that participate in the anaerobic cobalamin biosynthesis pathway are complete in all the analyzed GSB genomes, while some specific genes are absent in the aerobic cobalamin biosynthesis pathway. It was reported that symbiotic bacteria can provide vitamin B_12_ for algae (Croft et al., 2005) or B vitamins for insects (Salem et al., 2014). Corals and associated symbiotic Symbiodiniaceae are deficient in vitamin synthesis (Peixoto et al., 2021). High vitamin B_12_ concentrations and high bacteria abundances were found in gastric-cavity fluid of *G. fascicularis* (Agostini et al., 2012), and genes for vitamin B_12_ synthesis were found in some coral associated bacteria (Robbins et al., 2019), suggesting vitamin B_12_ may play an important role in the symbiotic relationship between corals and microbial partners. Therefore, the capability of anaerobic production of vitamin B_12_ may help CAP clade strains achieve a symbiotic relationship with corals.

Bacteriochlorophyll is important for phototrophic bacteria and different bacteriochlorophylls may enable bacteria to adapt to variable light intensity for photosynthesis. GSB synthesize bacteriochlorophylls (BChls) *a*, *b*, *c*, *d*, or *e* to assemble chlorosomes for light harvesting (Imhoff, 2014). In BChl *c-* and *d-* containing *Chlorobium vibrioforme* strain NCIB 8327 (Huster and Smith, 1990), the ratio of BChl *c*/BChl *d* could increase at low-light intensities (Saga et al., 2003). Green and brown color are two major morphotypes of GSB, and it was found that compared with green-colored BChl *c*-containing *Chlorobium* spp., only brown-colored BChl *e*-containing *Chlorobium* spp. were able to grow at low-light intensities (Borrego and Garciagil, 1995). Another study suggested that the last common ancestor of Chlorobiaceae belonged to the brown form, that was capable to synthesize BChl *e* and carotenoid isorenieratene and adapted to the low-light conditions (Grouzdev et al., 2022). In GSB, CruB is a γ-carotene cyclase producing isorenieratene and was only found in brown-colored GSB, while CruA, a paralog of CruB, is a lycopene monocyclase and was found in all GSB (Maresca et al., 2007, 2008; Grouzdev et al., 2022). Analysis of the bacteriochlorophyll synthesis pathway showed that all the analyzed GSB contained genes for producing Bchls *a*, *b*, *c*, and *d* but occasionally contained the gene *bciD*, which converts Bchl *c* to Bchl *e* (Supplementary Figure S5). Co-occurrence of *bciD* with *cruB* in *Prosthecochloris* was also observed, consistent with the previous report (Grouzdev et al., 2022). Among the CAP genomes, only *Candidatus Prosthecochloris* sp. N2 (abbreviated as PsN2) possess the capacity to produce Bchl *e* and isorenieratene. Another strain *Candidatus Prosthecochloris* sp. N1 (abbreviated as PsN1) was obtained with PsN2 from the same coral and could not produce Bchl *e* (Chen et al., 2021b). The major difference between PsN1 and PsN2 was that green-colored PsN1 inhabited the upper layer in skeleton and brown-colored PsN2 inhabited the deeper layer in the skeleton (Chen et al., 2021b). Therefore, different pigments may enable CAP strains to adapt to different microniches in the coral skeleton.

#### 3.3.2. Carbon metabolism

For carbon metabolism, nearly complete reductive citrate cycle module encoding genes, except *pycA* (pyruvate carboxylase subunit A), were identified in all *Prosthecochloris* genomes (Figure 3; Supplementary Figure S6), confirming the carbon fixing capability of *Prosthecochloris*. Meanwhile, *cooS* encoding carbon-monoxide dehydrogenase and *cooC* encoding CO dehydrogenase maturation factor were mainly identified in the CAP clade, with one exception of the absence of *cooS* in PsN2 which is due to incomplete genome assembly. Further analysis found that *cooS* and *cooC* are located within a single genomic locus, a putative operon between the *Prosthecochloris* conserved genes *pckG* (phosphoenolpyruvate carboxykinase) and *mrpA* (Na^+^/H^+^ antiporter subunit A; Figure 4A). This operon also encodes an additional C-type cytochrome and NitT/TauT family transport system including substrate−binding protein, permease protein and ATP − binding protein. Furthermore, many mobile genetic elements (MGEs) such as restriction modification (RM) systems, toxin-antitoxin (TA) systems and recombination-related gene *recQ*, are located directly downstream of this operon, suggesting that this genomic locus undergoes active genome recombination. This genome recombination may also cause the loss of the *cooS* operon in non-CAP genomes, leading to the differentiation of CO metabolic capacities.

Carbon monoxide is the simplest oxocarbon generated by photooxidation of dissolved organic matter (Conrad et al., 1982). Usually, protons are the final electron acceptors in the CO oxidation pathway (CO + H_2_O↔CO_2_ + H_2_), but some microorganisms can oxidize CO with elemental sulfur as an electron acceptor (Sorokin et al., 2022). *Rhodospirillum rubrum*, a purple nonsulfur photosynthetic bacterium, has *cooS* which enables this bacterium to use CO as a sole energy source during anaerobic growth in darkness (Schultz and Weaver, 1982). The presence of carbon monoxide dehydrogenase may help CAP strains to use the CO in the coral skeleton, and generated CO_2_ can also be fixed through the reductive citrate cycle. Measurement of CO concentrations in coral skeletons or using isotope labeled carbon are needed for further investigation to determine the importance of CO metabolism in coral skeletons.

#### 3.3.3. Nitrogen metabolism

For nitrogen metabolism, all *Prosthecochloris* genomes encode nitrogenase NifDKH for nitrogen fixation, generating ammonia (Figure 3). The major difference between CAP and non-CAP genomes in nitrogen metabolism based on KEGG orthology is the distinct distribution of carbonic anhydrase, which is involved in the transformation of two important nitrogen-containing compounds, carbamate and cyanate. Specifically, *Prosthecochloris* genomes encode three carbonic anhydrases (CAs) with different distribution patterns. γ-CA-1 is conserved in all *Prosthecochloris* genomes, while γ-CA-2 and β-CA are exclusively present in CAP genomes. CA catalyzes the interconversion of carbonic acid and carbon dioxide (HCO_3_^−^ + H^+^ < => CO_2_ + H_2_O). This capacity is critical for aquatic photoautotrophs to maintain productivity at ambient concentrations of CO_2_ by concentrating CO_2_ (Kaplan and Reinhold, 1999). At present, three classes of CAs (α, β and γ) were found in bacteria and they share low sequence similarities (Capasso and Supuran, 2015; Supuran and Capasso, 2017). Bacterial α-CAs usually contain an N-terminal signal sequence and possess a periplasmic or extracellular localization and β-CAs and γ-CAs were mostly found in cytoplasm (Marcus et al., 2005; Dobrinski et al., 2010; Gai et al., 2014; Capasso and Supuran, 2015). Bacterial CAs also differs in the catalytic efficiency and the equilibrium toward the formation of CO_2_ or HCO_3_^−^ (Gai et al., 2014; Capasso and Supuran, 2015). In general, bacterial α-CAs have higher catalytic efficiency than that of β-CAs which in turn are more efficient than γ-CAs (Capasso and Supuran, 2015). In *Ralstonia eutropha*, it was proposed that the periplasmic α-CA converts diffused CO_2_ to HCO_3_^−^ and may be responsible for supplementation of cellular bicarbonate, while one cytoplasmic γ-CA converts HCO_3_^−^ to CO_2_ and may be responsible for the supplementation of CO_2_ to RuBisCO, and one β-CA may function in pH homeostasis (Gai et al., 2014). Furthermore, different types of CAs were found in photosynthetic purple bacteria, that were able to synthesize CAs under both photoautotrophic and photoheterotrophic conditions (Ivanovsky et al., 2020). The diurnal rhythm of coral endosymbiont photosynthesis may lead to different availability of CO_2_ in skeletons for endolithic *Prosthecochloris.* During the day, dominant by photosynthesis of coral, the pH inside of coral skeletons reached over 8.5 and the major form of inorganic carbon is HCO_3_^−^ but not CO_2_ (Shashar and Stambler, 1992)_._ During the night, dominant by respiration of coral, pH decreased to approximately 7.7 (Shashar and Stambler, 1992). Therefore, the availability of HCO_3_^−^ or CO_2_ in coral skeletons for coral endolithic *Prosthecochloris* at night may different with that in the daytime. It is proposed that different CAs in *Prosthecochloris* genomes may play different roles in the utilization of CO_2_. Furthermore, considering that many pumps can deliver HCO_3_^−^, which is also important for coral calcification, it is a straightforward speculation that CAs in CAP genomes may facilitate coral calcification.

#### 3.3.4. Sulfur metabolism

Comparing all the known KEGG pathways of *Prosthecochloris* genomes, the numbers of modules (≥80% completeness) in sulfur metabolism were higher in CAP genomes (Supplementary Figure S7). Further analysis showed that the numbers of genes involved in assimilatory sulfate reduction (ASR), dissimilatory sulfate reduction (DSR) and thiosulfate oxidation by SOX complex were significantly higher in CAP genomes than that in non-CAP genomes (Supplementary Figure S8). ASR and DSR are the classical sulfate-reducing pathways that are involved in converting between sulfate and sulfide, with cysteine and sulfide as end products, respectively. For the ASR, *sat*, *cysND* and *cysC* are present in CAP genomes, while these genes are only present in a few non-CAP genomes (Figure 3). For DSR, *dsrAB* (dissimilatory sulfite reductase)*, sat* (sulfate adenylyltransferase) and *aprAB* (adenylylsulfate reductase) are present in CAP genomes, while *sat* and *aprAB* are absent in non-CAP genomes. The DSR pathway can convert sulfide to sulfate (Grein et al., 2013), and the lack of *sat* and *aprAB* genes suggests that non-CAP genomes can only convert the sulfide to sulfite (Supplementary Figure S9A). Analysis of the genomic loci of these genes revealed that the genes involved in DSR are located in a single genomic locus, while genomic regions containing *sat* and *aprAB* undergo genome rearrangement, which may lead to the loss of *sat* and *aprAB* genes (Supplementary Figure S9B).

Similarly, for thiosulfate oxidation by SOX complex, *soxYZ soxAX* and *soxB* are present in CAP genomes, while *soxAX* and *soxB* are absent in non-CAP genomes (Figure 3). In SOX complex, SoxYZ is responsible for substrate binding, the manganese-containing SoxB is responsible for hydrolyzing cysteinyl *S*-thiosulfonate to cysteinyl persulfide and sulfate, and SoxAX is a heterodimeric c-type cytochrome mediating electron transfer (Sakurai et al., 2010). Although SoxYZ homologs are present in both CAP and non-CAP genomes, they shared low amino acid sequence identity (~30%), suggesting different evolutionary sources of SoxYZ in *Prosthecochloris*. In CAP genomes, the SOX complex related genes *soxFXYZABW,* along with a porin-encoding gene and *dsbD* (cytochrome c-type biogenesis protein), apparently consist of an operon that was inserted in a genomic region conserved in all *Prosthecochloris* genomes (Supplementary Figure S9C). In non-CAP genomes, *soxYZ* are located adjacent to *fccAB* (sulfide dehydrogenase) forming the *soxYZ*-*fccAB* cluster. The gene cluster *soxYZ*-*fccAB* is conserved in other GSB (Gregersen et al., 2011). However, this *soxYZ* are absent in CAP genomes, leaving only *fccAB* genes (Supplementary Figure S9C). Furthermore, although the non-CAP genome *Prosthecochloris* sp. CIB 2401 (abbreviated PsCIB2401) has the *soxFXYZABW* gene cluster, it is located at a different genomic locus. BlastP searching using these two SoxF homologs found hits mostly from Gammaproteobacteria or sulfur-oxidizing bacteria (e.g., *Thiothrix*), consistent with the analysis of the *sox* gene cluster in another GSB *Chlorobium phaeovibrioides* DSM 265 (Gregersen et al., 2011). These results indicate that SOX complex related genes in *Prosthecochloris* genomes may be acquired by horizontal gene transfer (HGT) with subsequent gene loss events.

GSB typically oxidize sulfide and thiosulfate to sulfate, with extracellular sulfur globules as an intermediate, formed by incomplete oxidation of sulfide (Frigaard and Dahl, 2009; Holkenbrink et al., 2011). Oxidizing sulfide can provide GSB electrons for carbon fixation. It was found that the DSR system is required for sulfur globule oxidation in GSB, but is dispensable in environments with sufficiently high sulfide concentrations (Holkenbrink et al., 2011). Extracellular sulfur globules were observed in enrichment culture 2 dominated by SCSIO W1103 (Figure 1B), confirming that genes involved in sulfur metabolism are active in SCSIO W1103. PsC10, which shared the last common ancestor with the CAP clade, has the DSR pathway (Figure 3). Therefore, it is proposed that the last ancestor of *Prosthecochloris* can metabolize sulfur through assimilatory sulfate reduction, dissimilatory sulfate reduction and thiosulfate oxidation. However, genomic loci involved in sulfur metabolism undergo genome rearrangement leading to the evolutionary divergence of different sulfur metabolic capacities between CAP and non-CAP strains to adapt to sulfide-limiting habitats.

#### 3.3.5. *cbb*_3_-type cytochrome *c* oxidases and gas vesicles

Further analysis of modules in KEGG pathways revealed that genes encoding *cbb*_3_-type cytochrome *c* oxidases (*cbb*_3_-C*c*Os) are present in CAP genomes but not in non-CAP genomes (Figure 3; Supplementary Figure S8). Similar to the well-studied *cbb*_3_-C*c*Os in *Pseudomonas aeruginosa* PAO1, which contains two independent *ccoNOQP* operons, *cbb*_3_-C*c*O operons in CAP genomes have all the *ccb*_3_-C*c*O subunit genes *ccoNOQP* and *ccb*_3_-C*c*O maturation genes *ccoGHIS* with an additional conserved gene encoding putative sulfite exporter *tauE*. Unlike the *cbb*_3_-C*c*Os in strain PAO1, this *cbb*_3_-C*c*O operon contains an additional gene encoding a remote homolog of CcoH, and the CcoP protein (138 aa) in GSB is only half the length of that in strain PAO1 (319 aa). Further analysis of domain architectures showed that CcoP_GSB_ has only one Cyochrome_CBB3 domain (PF13442), while CcoP_PAO1_ has two Cyochrome_CBB3 domains (Figure 4B). CcoH is essential for *cbb*_3_-C*c*O assembly and interacts with CcoP primarily *via* interactions with the single transmembrane span of CcoH (Pawlik et al., 2010). Therefore, the GSB-derived *cbb*_3_-C*c*O may be different from classical *cbb*_3_-C*c*O in assembled enzymes. Similar to the *cooS* operon, *cbb*_3_-C*c*O operons are located in the conserved genomic loci of *Prosthecochloris* and were inserted with variable MGEs including TA systems (Figure 4B), suggesting that MGEs may be involved in the evolution of the *cbb*_3_-C*c*O operon. Furthermore, according to the phylogenetic tree and distribution of *cbb*_3_-C*c*O operon, it is proposed that *cbb*_3_-C*c*O operon was lost in non-CAP genomes due to environments diversification.

Different multiple terminal oxidases in bacterial respiratory chains can help bacteria to adapt to different environmental O_2_ concentrations. The *cbb*_3_-C*c*Os are important for microaerobic respiration, being essential for important nitrogen-fixing endosymbionts and for some human pathogens (Kulajta et al., 2006; Xie et al., 2014). In pathogens, *cbb*_3_-C*c*Os support aerobic respiration, and are also involved in denitrification of *P. aeruginosa* PAO1 and nitrite reduction of *Neisseria gonorrhoeae* under oxygen-limited conditions (Hopper et al., 2009; Hamada et al., 2014), as a strategy for pathogens to adapt to hypoxia during infection. In *Rhodobacter* group bacteria, photosynthetic bacteria that can grow under both anaerobic and aerobic conditions, *cbb*_3_-C*c*O can repress photosynthesis gene expression, as a regulator under different oxygen conditions (Kim et al., 2007; Ekici et al., 2012; Wang et al., 2014). In *Bradyrhizobium japonicum*, a nitrogen fixing bacterium, *cbb*_3_-C*c*O can protect nitrogenase from oxygen inactivation (Page and Guerinot, 1995). Oxygen levels in coral skeletons showed a diurnal rhythm (Kuhl et al., 2008). O_2_ concentrations increased in the daytime due to photosynthesis of endolithic algae, while greatly reduced at night. Although GSB are thought to be strictly anaerobic photoautotrophs, the presence of the *cbb*_3_-C*c*O in CAP genomes may enable CAP strains to detoxify oxygen and adapt to dynamic conditions to regulate photosynthesis and nitrogen fixing processes.

Further analysis of genes that were not included in the KEGG pathways revealed that gene clusters of gas vesicle proteins (*gvp*) were found in CAP genomes, which is consistent with our observation of gas vesicles in cells (Figure 1). Similar to the *gvp* gene cluster in *Serratia* sp. ATCC 39006, the *gvp* gene cluster in CAP genomes contains structural genes *gvpA* and *gvpC*, accessory genes and regulatory genes (Figure 4C). Additionally, this gene cluster contains genes encoding an apolipoprotein and a myocilin-like protein, a component of a membrane-associated protein complex. Comparing the upstream and downstream genes of the *gvp* gene cluster revealed that genome rearrangement may cause the loss of *gvp* gene cluster in non-CAP genomes, with one exception that the *gvp* gene cluster was rearranged to another genomic locus in PsCIB2401 (Figure 4C).

Many bacteria and archaea can produce gas vesicles, providing cells with buoyancy to maintain a suitable depth in the aqueous environment (Pfeifer, 2012; Tashiro et al., 2016). In cyanobacteria, the gas vesicles and the carbohydrates produced by photosynthesis serve as buoyancy and ballast to enable diurnal vertical migrations. This can help cells maintain photosynthesis and avoid floating to the ocean surface, where ultraviolet light may have a strong DNA-damaging effect on cells (Walsby, 1994). Similarly, the green sulfur bacterium *Pelodictyon phaeoclathratiforme* (now recognized as *Chlorobium clathratiforme*) also uses such a strategy when the light intensities are too high (Overmann et al., 1991). Although it is thought that gas vesicles are not used for gas storage, gas vesicles are permeable to oxygen, nitrogen, hydrogen, carbon dioxide, carbon monoxide and methane (Walsby et al., 1992). It was reported that different *Prosthecochloris* strains can form separated layers at different depths of the coral skeleton (Chen et al., 2021b). Additionally, the diurnal changing of CO_2_ and O_2_ concentration in coral skeletons may alter the positions of optimal micro-niches for *Prosthecochloris*. Considering that flagellar or gliding related genes were not present in CAP genomes, the presence of the *gvp* gene cluster in CAP genomes may enable cells to position inside the coral skeleton.

Moreover, CAP genomes encode 2–5 homologs of CphA (cyanophycin synthetases), while most non-CAP genomes lack CphA (Figure 3). Cyanophycin is a proteinogenic biopolymer that is composed of mainly arginine-aspartate dipeptides and is naturally produced by cyanobacteria. Cyanophycin serves as transient storage for extra nitrogen, carbon and energy (Ziegler et al., 1998), suggesting that CAP strains may store extra nutrients for survival in changing niches.

### 3.4. Differentiation of cell surface polysaccharides and phage susceptibility may drive the evolution and divergence within CAP clade genomes

**Figure 5:**
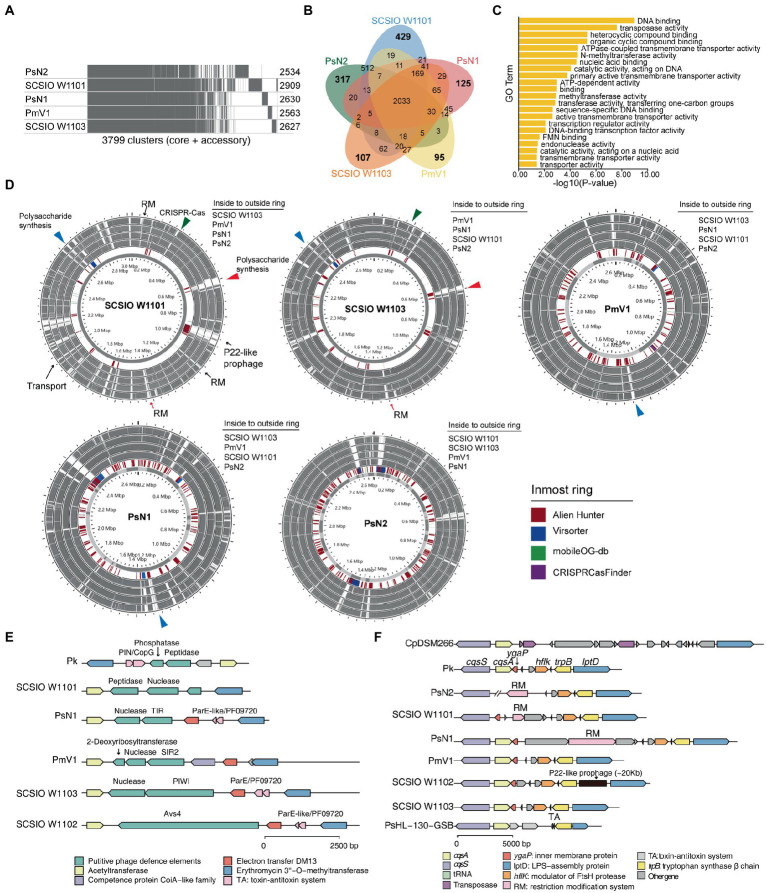
Polysaccharide synthesis gene cluster and phage defense systems drive the divergence and evolution within coral-associated *Prosthecochloris* (CAP) genomes. **(A)** Pangenome analysis of five CAP genomes. Considering the genome quality of MAGs, *Candidatus Prosthecochloris* korallensis and *Candidatus Prosthecochloris* sp. A305 and *Candidatus Prosthecochloris* sp. SCSIO W1102 were not included. Numbers at each line indicate the CDS numbers of each genome. A total of 3,799 gene clusters were found. **(B)** Venn diagrams of shared and specific genes among five CAP genomes. **(C)** Enrichment of *Candidatus Prosthecochloris* sp. SCSIO W1101 specific genes based on GO annotation of molecular functions (MF). **(D)** Genome alignment of these five genomes. Each genome was used as the reference, and MGE related regions predicted by different tools are indicated in the inmost ring. The same colored arrows or triangles indicate aligned variable genomic regions in different genomes. **(E)** Different phage defense systems are inserted in the conserved genomic regions in different genomes. **(F)** The absence of *cqsA* in the SCSIO W1101 genome may be caused by the integration or excision of MGEs.

We observed different cell morphologies between SCSIO W1101 and SCSIO W1103, although we have shown that CAP specific features are conserved within CAP genomes. Therefore, we questioned what is the difference between these highly similar genomes. Pangenome analysis showed that these five CAP genomes have 2033 conserved genes, and there were more specific genes in PsN2 or SCSIO W1101 than in *Candidatus Prosthecochloris* sp. N1 (abbreviated as PsN1), PmV1 or SCSIO W1103 (Figures 5A,B), consistent with the pairwise ANI among these genomes (Figure 2B). GO enrichment analysis of 429 specific genes of SCSIO W1101 showed that the functions of these genes are related to processes of DNA binding, transposase activity, nucleotide metabolism and transporters (Figure 5C; Supplementary Figure S10). Comparing these genomes using each genome as the reference, these variable genomic loci showed a mosaic pattern and were conserved in only one or a few genomes, and most of these were derived from MGEs, such RM systems, CRISPR–Cas systems and prophages (Figure 5D). The SCSIO W1101 genome contains a P22-like prophage that is absent in other genomes and specifically contains genes encoding an energy-coupling factor transport system consisting of conserved modules (EcfA: ATP-binding protein; EcfT: permease protein; HtsT: substrate-specific component; Figure 5D), which is involved in the uptake of vitamins in prokaryotes (Rodionov et al., 2009). Additionally, polysaccharide synthesis gene clusters are the major specific genes in SCSIO W1101, which was predicted as HGT from other bacteria by Alien Hunter (Vernikos and Parkhill, 2006). Some of the genes in the polysaccharide synthesis gene clusters were also found in other CAP genomes, suggesting that these partially conserved polysaccharide synthesis gene clusters may undergo rapid evolution. These results suggest that these CAP strains may have variable cell surface structures, consistent with our observed different cell morphologies.

Further analysis revealed a genomic hotspot in CAP genomes inserted by variable phage defense systems. This genomic locus is located between conserved genes encoding acetyltransferase and erythromycin 3”-O-methyltransferase (Figure 5E). Compressive annotation of these genes found that most of these genes encode MGEs including TA systems and nucleases. Among them, the gene in SCSIO W1102 encodes an antiviral STAND (Asv4)-like protein, a recently identified phage defense system that belongs to nucleotide-binding oligomerization domain-like receptors (Gao et al., 2022). Similarly, the gene in SCSIO W1103 encodes a short prokaryotic Argonaute system (containing a PIWI domain) with a nuclease as the effector, which was also a phage defense system identified recently (Koopal et al., 2022). Furthermore, genes encoding the SIR2 or TIR domain were identified in PmV1 and PsN1. SIR2 and TIR domains were demonstrated as part of phage defense systems (Ofir et al., 2021; Garb et al., 2022; Millman et al., 2022), which can both generate the signal molecule cyclic ADP-ribose analog once infected by phages, activating their associated effectors and leading to an abortive infection (Abi). Therefore, the TIR associated nuclease and SIR2 associated 2-deoxyribosyltransferase and nuclease may be putative effectors of phage defense systems in PmV1 and PsN1. Furthermore, the genes encoding putative phosphatase and peptidase were identified in SCSIO W1101 and *Candidatus Prosthecochloris* korallensis (abbreviated as Pk). Variable phage defense systems were previously found to be inserted in the same genomic locus among highly similar genomes (Rousset et al., 2022). Therefore, phosphatase and peptidase in SCSIO W1101 and Pk may represent novel phage defense systems. These results suggest that the interaction of the *Prosthecochloris* population with phages occurs in the coral skeleton, which also drives the evolution of the *Prosthecochloris* population. These results also indicate that rapid evolutionary turnover of phage defense systems by MGEs may endow anti-phage activity against different phages among clonal CAP strains, similar to a previous finding that MGEs drive *Vibrio* resistance to phages in the wild (Hussain et al., 2021).

In addition to polysaccharide synthesis gene clusters and phage defense systems, quorum sensing (QS) system may be another potential factor that can drive the evolution within CAP genomes. QS system is a process of bacterial cell- to-cell communication based on small signaling molecules to coordinate social behavior including bioluminescence, virulence factor production, CRISPR–Cas system activity and biofilm formation (Pawlik et al., 2010; Hoyland-Kroghsbo et al., 2017; Mukherjee and Bassler, 2019). Acyl-homoserine lactone, AI-2, CAI-1 and diffusible signal factor (DSF) based QS systems in Gram-negative bacteria have been extensively studied (Papenfort and Bassler, 2016). Only the CAI-1 QS system was found in GSB genomes (Figures 3, 5F). The *cqsA*/*cqsS* gene pair is always located adjacent to each other. CqsA is the CAI-1 autoinducer synthase, and CqsS is the CAI-1 autoinducer sensor kinase/phosphatase. Alignment of all the genomes revealed that the *cqsA*/*cqsS* gene pair was located in a conserved genomic locus, while the *cqsA* gene was lost in PsN2 and SCSIO W1101. We further confirmed that the loss of *cqsA* in SCSIO W1101 is real and that the loss of *cqsA* in PsN2 is caused by incomplete genome assembly (Figure 5F). Moreover, genes encoding transposases, tRNA, RM systems, TA systems and prophages were inserted in this genomic locus, indicating this genomic locus is a hotspot for MGE integration and recombination, which may also lead to the loss of *cqsA* in SCSIO W1101. Synthesis of autoinducers is an energy-consuming process, and loss of *cqsA* and maintenance of *cqsS* can enable bacteria to sense signals in a population without consuming energy to produce signals. Signaling-deficient mutant strains were prevalent in the QS system harboring bacteria (Keller and Surette, 2006), which is an evolutionary consequence in clonal bacterial populations. Taken together, differentiated QS systems may also drive the strain-level evolution of CAP strains.

## 4. Conclusion

In this study, complete circular genomes in the CAP clade from *G. fascicularis* were obtained. Comparative genomic analysis revealed the difference between CAP and non-CAP genomes, including genes involved the specialized metabolic capacities (CO oxidation, CO_2_ hydration and sulfur oxidation), gas vesicles (vertical migration in coral skeleton), and *cbb*_3_-type cytochrome *c* oxidases (oxygen tolerance and regulation). Within the CAP genomes, variable polysaccharide synthesis gene clusters and phage defense systems may endow bacteria with differential cell surface structures and phage susceptibility. Furthermore, MGEs or evidence of MGE-related HGT were found in most of the genomic loci containing the above genes, suggesting that MGEs play an important role in the evolutionary diversification between CAP and non-CAP strains and within CAP clade strains. These findings are similar to a previous report that the endolithic *Ruegeria* population may adapt to skeletons through sulfur oxidation and swimming motility (Luo et al., 2021), suggesting that the capacities of sulfur oxidation and motility may be important for bacterial adaptation in coral skeletons. Nonetheless, how these different capacities are involved in the symbiotic relationship with corals still needs further study.

## Data availability statement

The datasets presented in this study can be found in online repositories. The names of the repository/repositories and accession number(s) can be found at: https://www.ncbi.nlm.nih.gov/, PRJNA668462.

## Ethics statement

The animal study was reviewed and approved by the Management Office of the Sanya National Coral Reef Nature Reserve (China) and the Department of Ocean and Fisheries of Hainan Province.

## Author contributions

KT and XW contributed to conception and design of the study. ZN, KT, and WW collected and analyzed the data. PW and YG contributed to the statistical analysis. YW provided the samples. KT wrote the first draft of the manuscript. XW, S-JK, and JY revised the manuscript. All authors contributed approved the submitted version.

## Funding

This work was supported by the National Science Foundation of China (91951203 and 42188102), National Key R&D Program of China (2022YFC3103600), Foundation Incubation Fund of Chinese Academy of Sciences (JCPYJJ-22025), Guangdong Basic and Applied Basic Research Foundation (2022A1515010702), the Open Project of State Key Laboratory of Marine Resource Utilization in South China Sea of Hainan University (MRUKF2021004), Science & Technology Fundamental Resources Investigation Program (2022FY100600), Local Innovative and Research Teams Project of Guangdong Pearl River Talents Program (2019BT02Y262), Guangdong Major Project of Basic and Applied Basic Research (2019B030302004) and the Youth Innovation Promotion Association CAS (2021345 to PW).

## Conflict of interest

The authors declare that the research was conducted in the absence of any commercial or financial relationships that could be construed as a potential conflict of interest.

## Publisher’s note

All claims expressed in this article are solely those of the authors and do not necessarily represent those of their affiliated organizations, or those of the publisher, the editors and the reviewers. Any product that may be evaluated in this article, or claim that may be made by its manufacturer, is not guaranteed or endorsed by the publisher.

## Supplementary material

The Supplementary material for this article can be found online at: https://www.frontiersin.org/articles/10.3389/fmicb.2023.1138751/full#supplementary-material

## Footnotes

^1^https://www.kegg.jp/blastkoala/    and    https://www.genome.jp/tools/kofamkoala/
