# Supplementary material for: Comparative genomic insights into habitat adaptation of coral-associated Prosthecochloris
Source: Front Microbiol. 2023 Apr 20;14:1138751. doi: 10.3389/fmicb.2023.1138751 (PMC10158934; doi:10.3389/fmicb.2023.1138751)
Supplement: Supplementary file 1 [file Data_Sheet_1.pdf]

Supplementary Material

Comparative genomic insights into habitat adaptation of coral-associated *Prosthecochloris*

Zhaolong Nie [1,2], Kaihao Tang [1,2,3]*, Weiquan Wang [2], Pengxia Wang [2,3,4], Yunxue Guo [2,3,4], Yan Wang [1], Shuh-Ji Kao [1], Jianping Yin[2], Xiaoxue Wang [2,3,4]

[1]State Key Laboratory of Marine Resource Utilization in South China Sea, Hainan University, Haikou 570228, China

[2]Key Laboratory of Tropical Marine Bio-resources and Ecology, Guangdong Key Laboratory of Marine Materia Medica, Innovation Academy of South China Sea Ecology and Environmental Engineering, South China Sea Institute of Oceanology, Chinese Academy of Sciences, Guangzhou 511458, China

[3] Southern Marine Science and Engineering Guangdong Laboratory (Guangzhou), Guangzhou 511458, China

[4]University of Chinese Academy of Sciences, Beijing 100049, China

***Correspondence** to: Kaihao Tang; Email: khtang@scsio.ac.cn

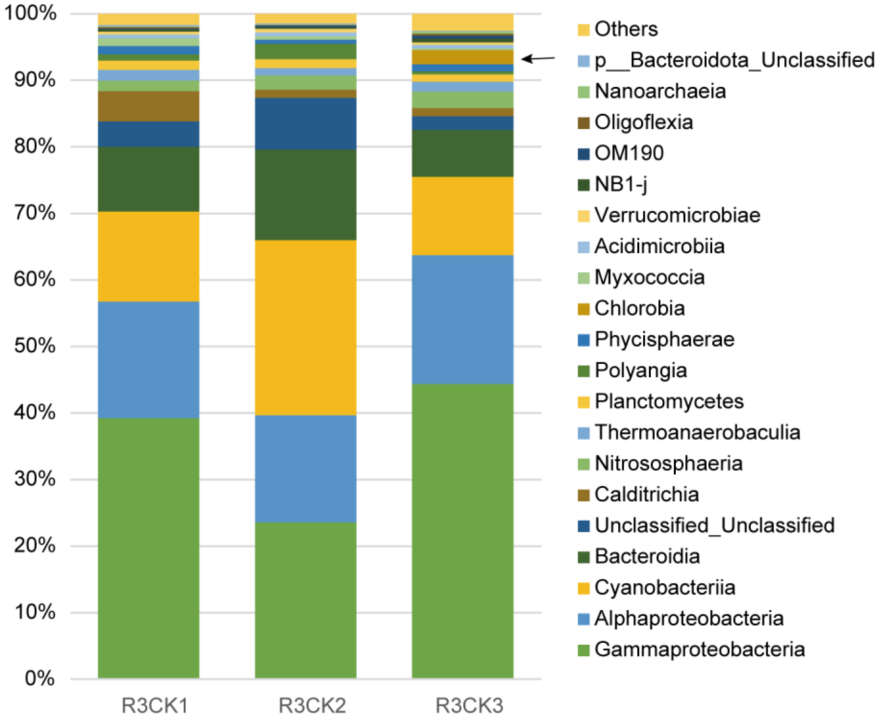

Figure S1. Green sulfur bacteria were detected in the stony coral *Galaxea fascicularis*. The amplicon sequencing data of 16S rRNA gene V4 region of three polype samples (R1CK1, R3CK2 and R3CK3) from our previous study (Wang et al., 2022) was reanalyzed. *Prosthecochloris* is the only genus in the phylum Chlorobi in these data.

PsCIB2401	100																																		
PsCIB2401	100	100																																	
PvDSM260	99.9	99.9	100																																
PvDSM260	99.9	99.9	100	100																															
PvM50B85	99.8	99.8	99.9	99.9	100																														
PvM55B161	99.8	99.8	99.9	99.9	100	100																													
PvM55B161_	98.5	98.5	98.6	98.6	98.7	98.7	100																												
DSM1685	97.9	97.9	97.8	97.8	97.7	97.7	96.5	100																											
PeN2_	97.9	97.9	97.8	97.8	97.7	97.7	96.5	100	100																										
PeN3_	97.9	97.9	97.8	97.8	97.7	97.7	96.5	100	100	100																									
PsvZM_2_	97.8	97.8	97.7	97.7	97.6	97.6	96.5	99.9	99.9	99.9	100																								
PsHL130GSB_	97.2	97.2	97.1	97.1	97.0	97.0	95.9	98.5	98.5	98.5	98.5	100																							
PsHL130GSB_	97.2	97.2	97.1	97.1	97.0	97.0	95.9	98.5	98.5	98.5	98.5	100	100																						
PsSM2_OrangeGreen1	97.3	97.3	97.2	97.2	97.1	97.1	95.9	98.6	98.6	98.6	98.5	99.9	99.9	100																					
PsTy1	97.1	97.1	97.0	97.0	97.0	97.0	95.9	97.7	97.7	97.7	97.6	98.3	98.3	98.4	100																				
PsTy1	97.1	97.1	97.0	97.0	97.0	97.0	95.9	97.7	97.7	97.7	97.6	98.3	98.3	98.4	100	100																			
PaDSM271	95.9	95.9	96.0	96.0	96.1	96.1	95.0	97.4	97.4	97.4	97.5	96.8	96.8	96.9	96.3	96.3	100																		
PsZM	95.9	95.9	95.9	95.9	95.9	95.9	94.8	97.3	97.3	97.3	97.4	96.8	96.8	96.9	96.3	96.3	99.8	100																	
PsB10	95.9	95.9	95.9	95.9	96.1	96.1	94.9	97.2	97.2	97.2	97.3	96.7	96.7	96.7	96.3	96.3	99.8	99.7	100																
PsC10	95.9	95.9	95.9	95.9	95.9	95.9	95.1	96.4	96.4	96.4	96.3	96.5	96.5	96.6	97.2	97.2	96.3	96.1	96.4	100															
Pk	96.8	96.8	96.7	96.7	96.7	96.7	95.9	97.1	97.1	97.1	97.0	96.6	96.6	96.7	97.1	97.1	96.5	96.4	96.6	97.5	100														
ASV195	96.5	96.5	96.6	96.6	96.6	96.6	96.1	97.0	97.0	97.0	96.9	96.3	96.3	96.3	96.3	96.3	96.3	96.2	96.4	96.7	98.6	100													
ASV399	96.5	96.5	96.5	96.5	96.4	96.4	96.1	96.9	96.9	96.9	96.8	96.3	96.3	96.3	96.2	96.2	96.0	96.0	96.2	96.6	98.6	100	100												
PsN2	96.9	96.9	96.9	96.9	96.8	96.8	96.1	97.1	97.1	97.1	97.0	96.5	96.5	96.5	96.7	96.7	96.3	96.4	96.5	96.7	98.5	99.2	99.1	100											
SCSIO W1101	96.9	96.9	96.8	96.8	96.7	96.7	96.0	97.1	97.1	97.1	97.1	96.5	96.5	96.5	96.7	96.7	96.4	96.5	96.5	96.7	98.7	99.4	99.3	99.9	100										
PsN1	96.9	96.9	96.8	96.8	96.7	96.7	96.0	97.1	97.1	97.1	97.1	96.5	96.5	96.5	96.7	96.7	96.4	96.5	96.5	96.7	98.7	99.4	99.3	99.9	100	100									
ASV4610	96.7	96.7	96.6	96.6	96.5	96.5	96.3	96.9	96.9	96.9	96.8	96.3	96.3	96.3	96.4	96.4	96.2	96.3	96.3	96.5	98.6	99.3	99.3	99.9	100	100	100								
SCSIO W1103	96.8	96.8	96.7	96.7	96.7	96.7	95.9	97.1	97.1	97.1	97.0	96.4	96.4	96.5	96.6	96.6	96.3	96.4	96.5	96.7	98.6	99.3	99.3	99.8	99.9	99.9	100	100							
SCSIO W1103	96.8	96.8	96.7	96.7	96.7	96.7	95.9	97.1	97.1	97.1	97.0	96.4	96.4	96.5	96.6	96.6	96.3	96.4	96.5	96.7	98.6	99.3	99.3	99.8	99.9	99.9	100	100	100						
PmV1	96.8	96.8	96.7	96.7	96.7	96.7	95.9	97.1	97.1	97.1	97.0	96.4	96.4	96.5	96.6	96.6	96.3	96.4	96.5	96.7	98.6	99.3	99.3	99.8	99.9	99.9	100	100	100	100					
ASV5361	96.6	96.6	96.7	96.7	96.7	96.7	96.2	96.9	96.9	96.9	96.8	96.2	96.2	96.3	96.4	96.4	96.3	96.4	96.5	96.5	98.6	99.4	99.3	99.9	100	100	100	99.9	99.9	99.9	100				
ClDSM245	93.5	93.5	93.6	93.6	93.5	93.5	92.5	92.8	92.8	92.8	92.8	93.4	93.4	93.5	94.0	94.0	93.1	93.1	93.0	93.3	93.1	92.4	92.7	92.9	92.8	92.8	92.7	92.8	92.8	92.8	92.3	100			
ClDSM245	93.5	93.5	93.6	93.6	93.5	93.5	92.5	92.8	92.8	92.8	92.8	93.4	93.4	93.5	94.0	94.0	93.1	93.1	93.0	93.3	93.1	92.4	92.7	92.9	92.8	92.8	92.7	92.8	92.8	92.8	92.3	100	100		
CpDSM266	93.5	93.5	93.4	93.4	93.4	93.4	92.4	93.4	93.4	93.4	93.4	93.7	93.7	93.7	94.2	94.2	93.9	93.8	93.8	93.9	93.6	93.2	93.4	93.7	93.6	93.6	93.4	93.5	93.5	93.5	93.1	96.6	96.6	100	
CpDSM266	93.5	93.5	93.4	93.4	93.4	93.4	92.4	93.4	93.4	93.4	93.4	93.7	93.7	93.7	94.2	94.2	93.9	93.8	93.8	93.9	93.6	93.2	93.4	93.7	93.6	93.6	93.4	93.5	93.5	93.5	93.1	96.6	96.6	100	100

Figure S2. Matrix of 16S rRNA genes sequence identity. Sequence names in blue color are derived from this study.

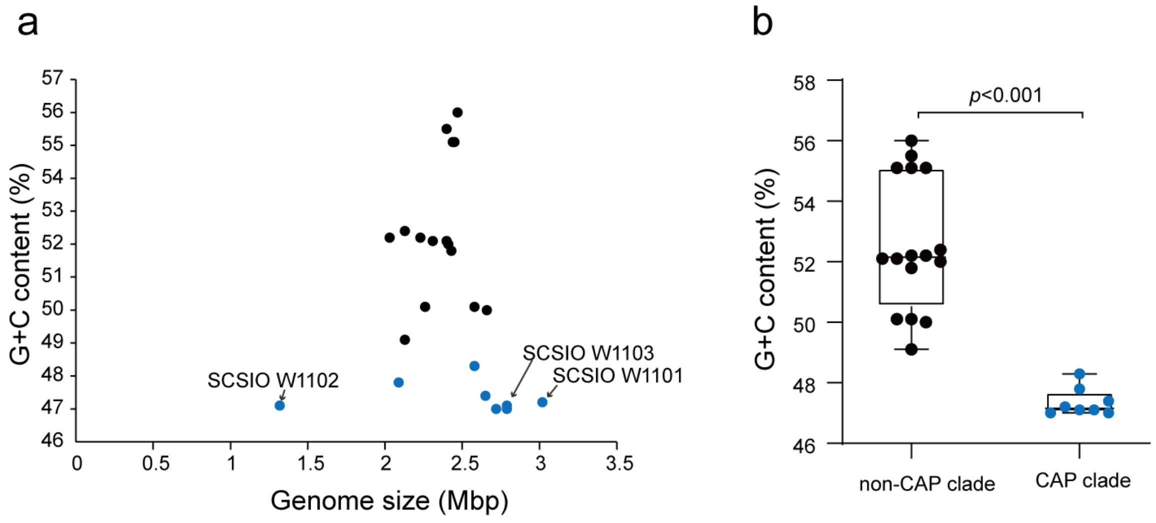

Figure S3. The CAP clade genomes have a significant lower G+C content. a, Scatter diagram of genome sizes and G+C contents of *Prosthecochloris* genomes. **b**, Comparison of G+C content between coral-associated *Prosthecochloris* (CAP) genomes and non-CAP genomes. Blue dots indicate the CAP genomes.

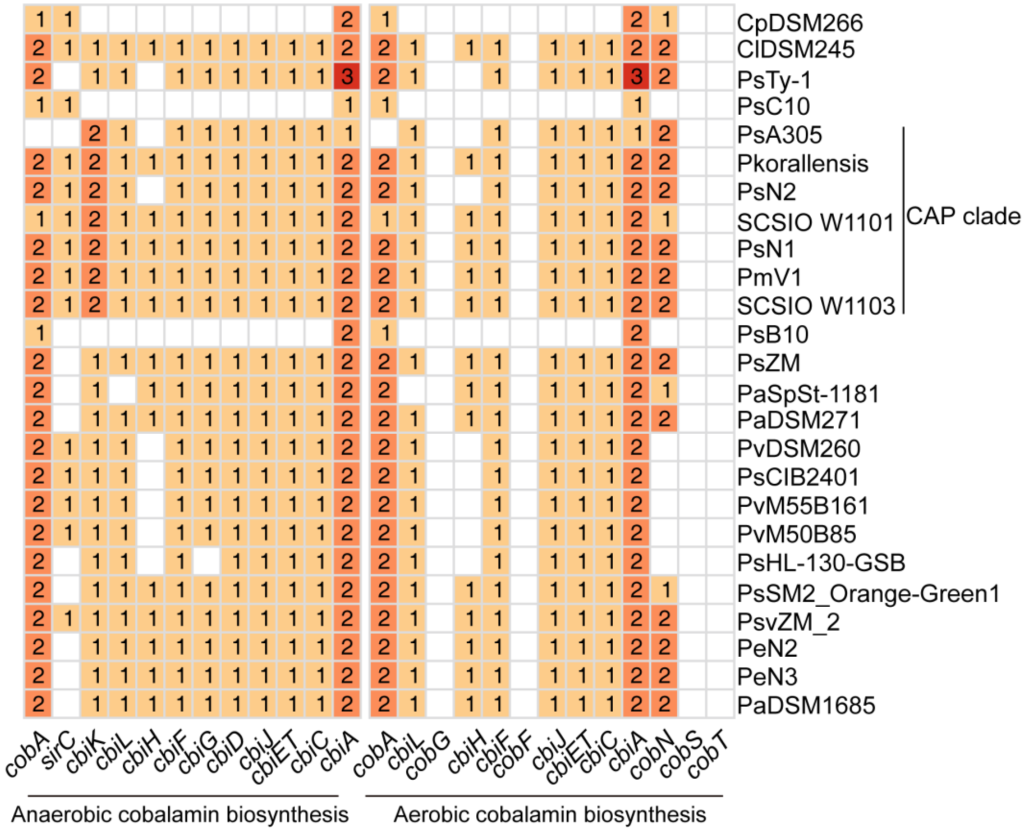

Figure S4. Presence and absence of genes involved in anaerobic and aerobic cobalamin biosynthesis pathways. Analysis was based on the KEGG orthology. Numbers in the cells indicate the numbers of genes predicted.

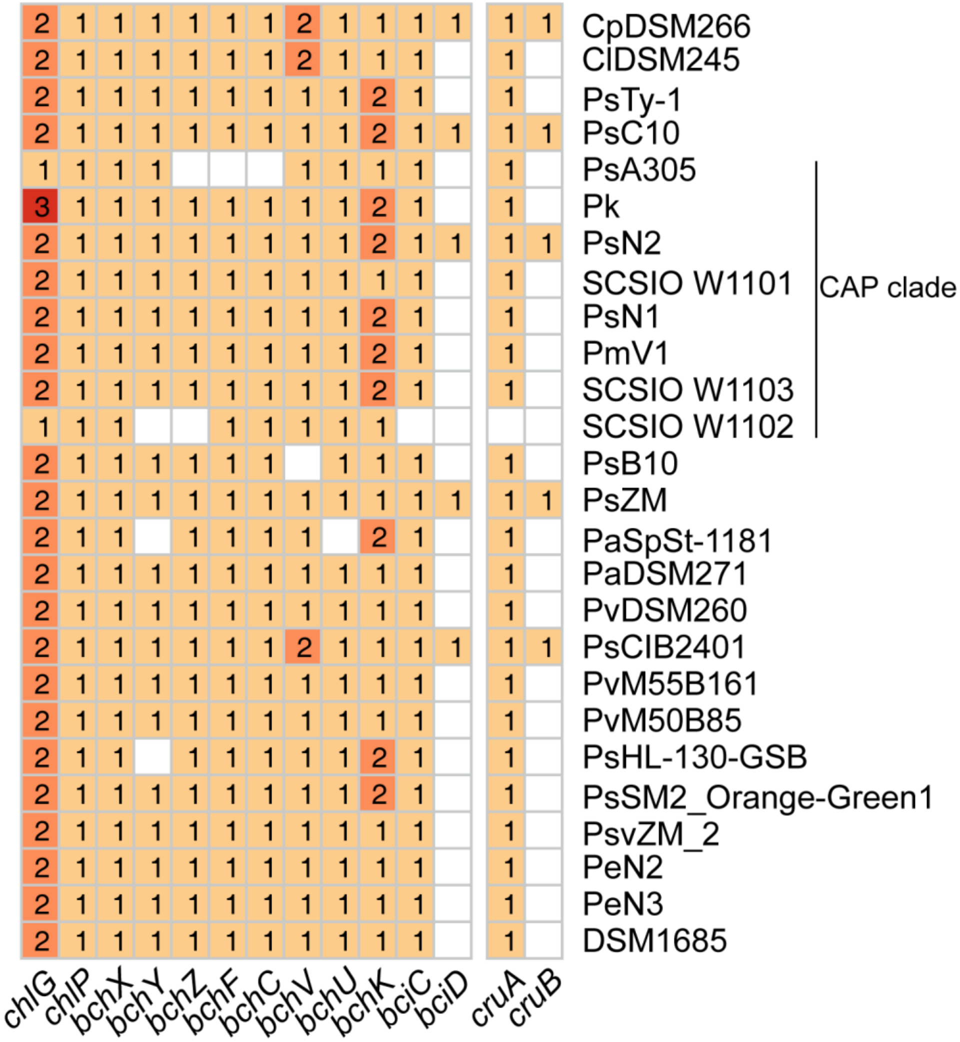

Figure S5. Presence and absence of genes involved in bacteriochlorophyll and carotenoid synthesis pathways. Analysis was based on the KEGG orthology. Numbers in the cells indicate the numbers of genes predicted.

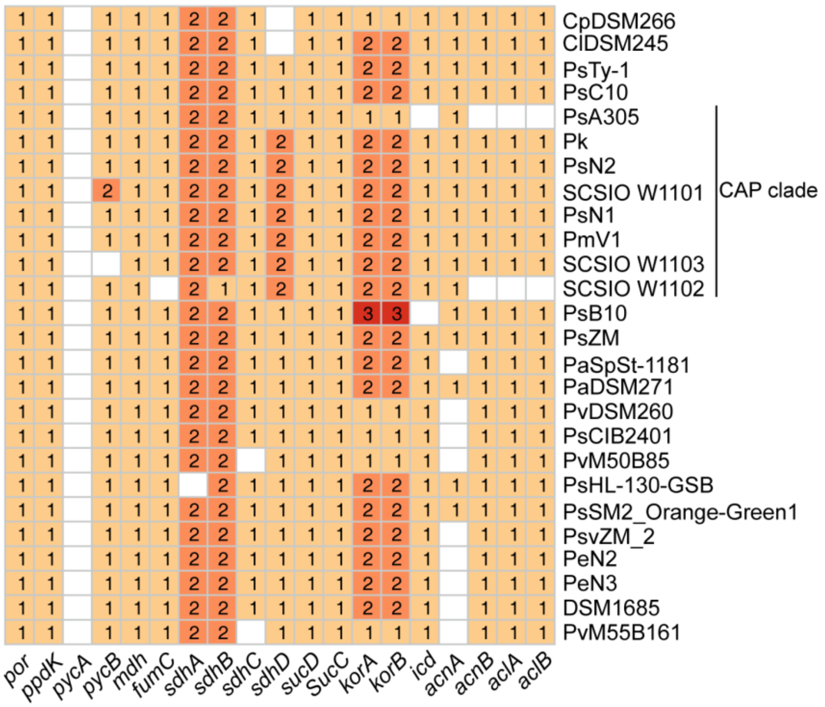

Figure S6. Presence and absence of genes involved in reductive citrate cycle. Analysis was based on the KEGG orthology. Numbers in the cells indicate the numbers of genes predicted.

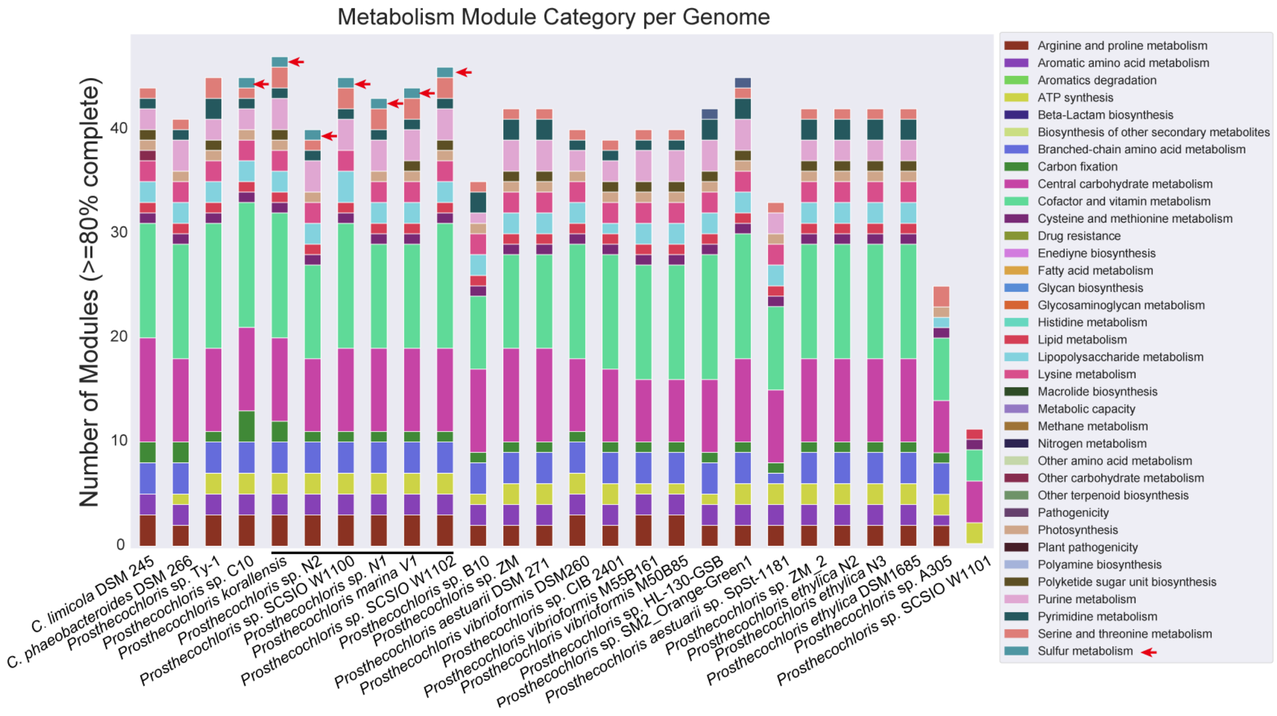

Fig S7. Metabolic summary based on KEGG pathways. Analysis was based on gene annotation by KofamKOALA using KEMET. The red arrow indicates the sulfur metabolism pathway.

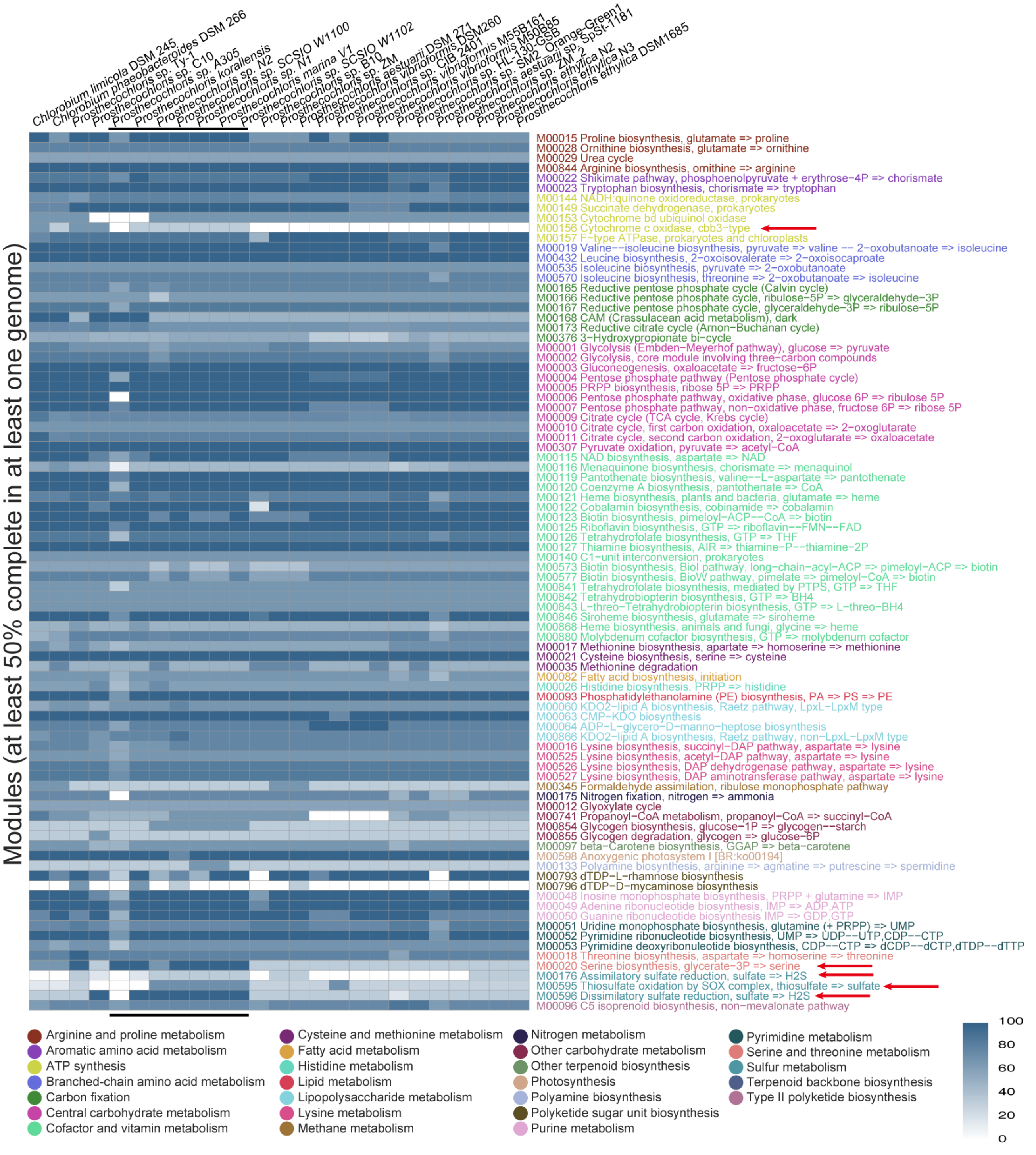

Figure S8. Comparison of metabolism modules of KEGG pathways. Analysis was based on gene annotation by KofamKOALA using KEMET. The red arrows indicate that completenesses of these modules are different between CAP and non-CAP genomes.

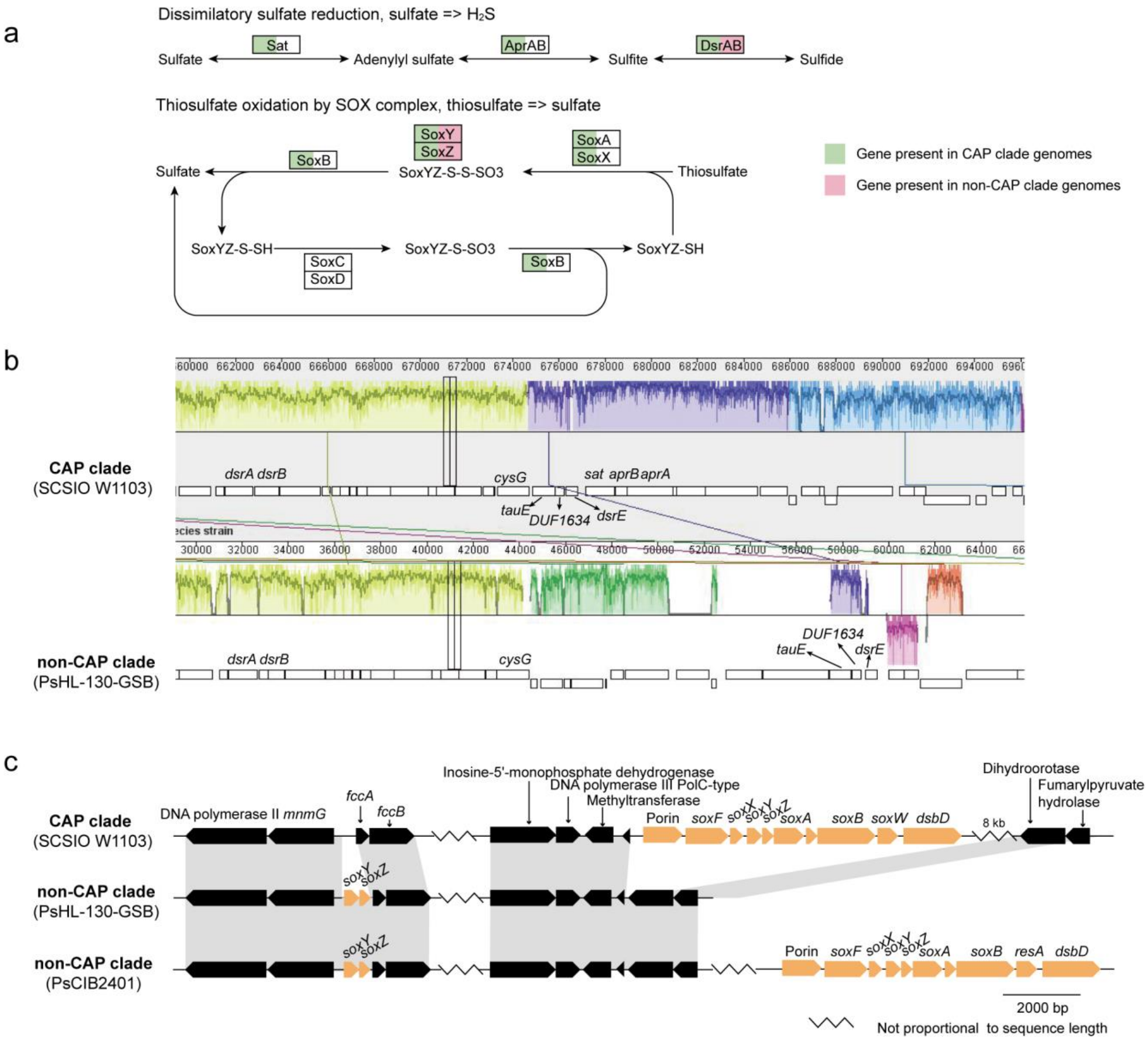

Figure S9. Presence and absence of genes involved in sulfur cycle. a, Presence and absence of genes involved in dissimilatory sulfate reduction and thiosulfate oxidation by SOX complex. **b,** Genetic maps of genes involved in dissimilatory sulfate reduction in coral-associated *Prosthecochloris* (CAP) genomes and non-CAP genomes. Genome sequences were aligned by Mauve. **c**, Location of two different SOX complex related gene cluster in CAP and non-CAP genomes. Gray shading indicates aligned genomic regions.

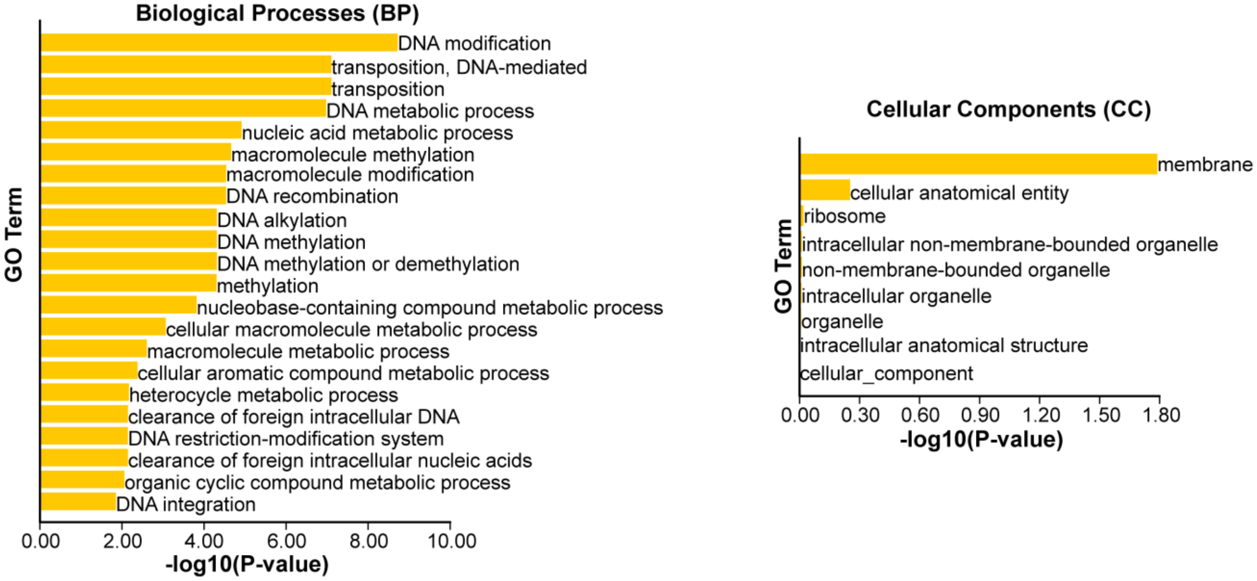

Figure S10. Enrichment of *Candidatus* Prosthecochloris sp. SCSIO W1101 specific genes based on GO annotation of biological processes (BP) and cellular components (CC).

Table S1. Assessment and classification of bins in enrichment culture metagenomes.

Bin Id	Assessment of quality and profile of bins by CheckM							Classification by GTDB-tk		
	Bin size (Mbp)	mapped reads	% mapped reads	% binned populations	% community	Completeness	Contamination	Classification	fastani_reference	fastani_ani
E1-1	3.67	296419	6.49	5.25	4.98	97.36	0.61	*Marinobacter hydrocarbonoclasticus*	GCF_000284615.1	98.47
E1-2	3.73	252523	5.53	4.4	4.17	98.71	0.57	*Halomonas piezotolerans*	GCF_012427705.1	98.59
E1-3	4.13	164192	3.59	2.58	2.45	87.07	1.77	f__Geopsychrobacteraceae; g__BM522	NA	NA
E1-4	3.37	1916569	41.94	36.98	35.05	92.73	16.48	*Prosthecochloris marina*	GCF_003182595.1	97.54
E1-5	2.16	1622896	35.51	48.87	46.32	81.19	4.95	*Prosthecochloris marina*	GCF_003182595.1	98.66
E1-6	2.69	79223	1.73	1.92	1.82	84.21	4.15	*Halodesulfovibrio* sp015482765	GCA_015482765.1	98.63
unbinned	2.28	238154	5.21	NA	5.21	NA	NA	NA	NA	NA
E2-1	5.26	1178217	15.03	9.77	8.94	99.86	0.11	*Vibrio alginolyticus*	GCF_000354175.2	98.41
E2-2	2.75	4011173	51.16	63.65	58.24	93.74	1.39	*Prosthecochloris marina*	GCF_003182595.1	98.96
E2-3	3.24	1487806	18.98	20.02	18.32	89.86	4.46	*Marinobacter hydrocarbonoclasticus*	GCF_000284615.1	98.33
E2-4	4.32	142665	1.82	1.44	1.32	99.43	1.37	*Halodesulfovibrio* sp.	NA	NA
E2-5	3.19	261218	3.33	3.58	3.27	89.86	4.46	*Halomonas piezotolerans*	GCF_012427705.1	99.15
E2-6	2.65	93633	1.19	1.54	1.41	32.56	5.77	*Halodesulfovibrio* sp.	NA	NA
unbinned	6.74	665876	8.49	NA	8.49	NA	NA	NA	NA	NA

NA: not available

Reference

Wang, W., Tang, K., Wang, P., Zeng, Z., Xu, T., Zhan, W., Liu, T., Wang, Y., and Wang, X. (2022). The coral pathogen *Vibrio coralliilyticus* kills non-pathogenic holobiont competitors by triggering prophage induction. *Nat Ecol Evol* 6**,** 1132-1144.
